# Binding of HIV-1 gp120 to DC-SIGN Promotes ASK-1-Dependent Activation-Induced Apoptosis of Human Dendritic Cells

**DOI:** 10.1371/journal.ppat.1003100

**Published:** 2013-01-31

**Authors:** Yongxiong Chen, Shiuh-Lin Hwang, Vera S. F. Chan, Nancy P. Y. Chung, Shu-Rong Wang, Zhongye Li, Jing Ma, Chia-Wei Lin, Ya-Ju Hsieh, Kao-Ping Chang, Sui-Sum Kung, Yi-Chia Wu, Cheng-Wei Chu, Hsiao-Ting Tai, George F. Gao, Bojian Zheng, Kazunari K. Yokoyama, Jonathan M. Austyn, Chen-Lung S. Lin

**Affiliations:** 1 The Eye Institute, Faculty of Medicine, Xiamen University, Xiamen, Fujian, China; 2 Department of Surgery, The University of Hong Kong Medical Center, Hong Kong, SAR, China; 3 Department of Surgery, Kaohsiung Medical University, Kaohsiung, Taiwan; 4 Institute of Microbiology, Chinese Academy of Science, Beijing, China; 5 Department of Microbiology, The University of Hong Kong Medical Center, Hong Kong, SAR, China; 6 Graduate School of Medicine, Kaohsiung Medical University, Kaohsiung, Taiwan; 7 Nuffield Department of Surgical Sciences, University of Oxford, Oxford, United Kingdom; University of Pennsylvania School of Medicine, United States of America

## Abstract

During disease progression to AIDS, HIV-1 infected individuals become increasingly immunosuppressed and susceptible to opportunistic infections. It has also been demonstrated that multiple subsets of dendritic cells (DC), including DC-SIGN(+) cells, become significantly depleted in the blood and lymphoid tissues of AIDS patients, which may contribute to the failure in initiating effective host immune responses. The mechanism for DC depletion, however, is unclear. It is also known that vast quantities of viral envelope protein gp120 are shed from maturing HIV-1 virions and form circulating immune complexes in the serum of HIV-1-infected individuals, but the pathological role of gp120 in HIV-1 pathogenesis remains elusive. Here we describe a previously unrecognized mechanism of DC death in chronic HIV-1 infection, in which ligation of DC-SIGN by gp120 sensitizes DC to undergo accelerated apoptosis in response to a variety of activation stimuli. The cultured monocyte-derived DC and also freshly-isolated DC-SIGN(+) blood DC that were exposed to either cross-linked recombinant gp120 or immune-complex gp120 in HIV(+) serum underwent considerable apoptosis after CD40 ligation or exposure to bacterial lipopolysaccharide (LPS) or pro-inflammatory cytokines such as TNFα and IL-1β. Furthermore, circulating DC-SIGN(+) DC that were isolated directly from HIV-1(+) individuals had actually been pre-sensitized by serum gp120 for activation-induced exorbitant apoptosis. In all cases the DC apoptosis was substantially inhibited by DC-SIGN blockade. Finally, we showed that accelerated DC apoptosis was a direct consequence of excessive activation of the pro-apoptotic molecule ASK-1 and transfection of siRNA against ASK-1 significantly prevented the activation-induced excessive DC death. Our study discloses a previously unknown mechanism of immune modulation by envelope protein gp120, provides new insights into HIV immunopathogenesis, and suggests potential therapeutic approaches to prevent DC depletion in chronic HIV infection.

## Author Summary

HIV-1 infected individuals become increasingly immunocompromised and susceptible to opportunistic infection during disease progression, which is associated with significant reduction of the dendritic cell number in the peripheral blood or secondary lymphoid tissues. Because dendritic cells are the most powerful antigen-presenting cells, their survival is critical for host defence and inadequate dendritic cell number will fail to induce effective host immune responses. Here we describe a mechanism that may at least partly explain why dendritic cells become significantly depleted in chronic HIV-1 infection. We found that after binding of the HIV-1 envelope protein gp120 to the dendritic cell surface protein DC-SIGN, the subsequent activation by CD40 ligation, or by exposure to bacterial product lipopolysaccharide or pro-inflammatory cytokines such as TNF-α and IL-1β, will lead to overexpression of pro-apoptotic molecule ASK-1, resulting in excessive dendritic cell death. We also confirmed that DC-SIGN(+) dendritic cells in the blood of HIV-1 infected individuals have actually been pre-sensitized by viral gp120, which exists in vast amount in the blood, for activation-induced exorbitant death. Our study thus reveals a previously unknown pathway for dendritic cell depletion and provides clues for potential therapeutic approaches to prevent DC depletion in chronic HIV infection.

## Introduction

HIV-1 envelope protein gp120 binds to CD4 and chemokine receptors CCR5 or CXCR4 which are expressed by dendritic cells (DC) and which facilitate viral entry into the cells [1]. HIV-1 gp120 is also readily shed from the maturing virions [2] and forms immune complexes in the plasma of HIV-infected [HIV(+)] individuals [3], [4]; consequently only a tiny portion (∼0.1%) of circulating virions are actually infectious [5], [6]. HIV-1 gp120 additionally binds to DC-specific ICAM-grabbing non-integrin (DC-SIGN), initiating an intracellular signalling cascade that promotes viral infection and dissemination to T cells [7], [8]. A subset of CD14(+)DC-SIGN(+) DC has been identified in blood, which can bind HIV-1 and to transmit infectious virus to T cells [9]. The virus then actively replicates in activated CD4 T cells, which are chronically induced during HIV infection by various mechanisms [10], [11].

During progression to AIDS, HIV(+) individuals become increasingly immunosuppressed and susceptible to opportunistic infections and some cancers. This is accompanied by progressive depletion of DC from different anatomical compartments, but the reasons for this remain largely unknown. For example, it has been demonstrated that by *in situ* hybridization, DC-SIGN expression was significantly reduced in the spleen of SIV-induced AIDS [12]. Furthermore, in late-stage HIV infection, a dramatic depletion of lymph node myeloid DC (mDC) was also observed, with mDC 20-fold less frequent in HIV(+) nodes than in control nodes [13]. Consistently, another report employed flow cytometry and immunofluorescence study to show that the frequencies of lymph node mDC were significantly decreased in a model of simian AIDS [14], suggesting that mDC are lost from rather than being recruited to lymphoid tissue in advanced SIV infection. In addition, mDC from SIV-infected animals undergo spontaneous cell death during culture [14], supporting the hypothesis that the loss of mDC may be due to cell death. Moreover, DC that are annexin V-positive could be identified in the lymph nodes of monkeys with AIDS, indicating that the DC in the LN may be undergoing apoptosis [14].

Under normal circumstances the life-span and availability of DC *in vivo* is crucial for the induction and maintenance of effective antigen-specific T cell immunity [15], [16]. For example, the magnitude and quality of the CD4 T cell response is proportional to the number of antigen-bearing DC that reach the lymph nodes [17]. Normally, migration of DC from peripheral non-lymphoid sites into secondary lymphoid tissues is promoted by DC maturation, the process by which the cells acquire enhanced capacities for T cell activation and the regulation of immune responses. In peripheral sites of infection and inflammation, DC maturation can be induced by stimuli such as bacterial lipopolysaccharide (LPS) and the pro-inflammatory cytokines TNF-α and IL-β. Within the lymphoid tissues, further DC activation can be induced by activated T cells that upregulate CD40 ligand (CD40L) which ligates CD40 on the DC [18]. DC then undergo apoptosis in a tightly-regulated process. Accumulating evidences have demonstrated that the balance between survival *versus* apoptosis of DC is controlled by differential expression of anti- and pro-apoptotic molecules that are induced during different cellular responses. For example, the above maturation stimuli can activate phosphatydylinositide 3-kinase (PI3K) which phosphorylates Akt (p-Akt) [19] and this, in turn, increases expression of anti-apoptotic proteins such as Bcl-2 and Bcl-xL [20], [21]. It has been further shown that binding of HIV-1 gp120 to DC-SIGN recruits effector proteins to the DC-SIGN signalosome to phosphorylate and activate Raf-1 (p-Raf) [22]. Raf-1 is anti-apoptotic and can in turn antagonize the function of another MAPKKK, apoptosis signal regulating kinase-1 (ASK-1) [23]. ASK-1 can also be activated (p-ASK-1) by the above stimuli but is pro-apoptotic, at least in part through its capacity to reduce expression of Bcl-2 and Bcl-xL [24]–[26]. Because the regulation of DC survival in peripheral and lymphoid tissues is crucial for the initiation and regulation of immune responses [15], any reduction in DC numbers would result in generalised immunosuppression and increased susceptibility to opportunistic and other infections, as is observed during disease progression to AIDS.

We previously reported that chemotaxis of monocyte-derived DC (moDC) can be induced by M-tropic HIV-1 (R5 strains) through binding of gp120 to CCR5 [27]. This finding may in part explain the observation that during acute HIV-1 infection there is rapid accumulation of DC-SIGN(+) cells within the lymphoid tissues [12]. In contrast, as disease progresses chronically to AIDS, circulating and lymphoid tissue-associated DC become progressively depleted [28]–[33]. These include DC-SIGN(+) DC which are markedly reduced in lymph nodes of AIDS patients [34] and in spleens of non-human primates with SIV-induced AIDS [12]. Clinical observations indicate that the increase in serum HIV-1 viral loads correlates well with the decrease in numbers of DC in HIV-1-infected individuals [29], [34]–[37], suggesting that the virus itself or viral products such as shed gp120 may directly impact on DC survival.

Much work has focussed on the mechanisms of immune subversion by infectious HIV-1 virions, but relatively scant attention has been paid to potential immunomodulatory effects of the vast amounts of gp120 immune complexes within the circulation of HIV(+) individuals. We hypothesised that binding of gp120 to DC-SIGN(+)DC may have an impact on their survival, thus contributing to the cellular depletion observed in the settings above. Here we show that DC which are exposed to HIV-1 gp120, either *in vitro* or *in vivo*, undergo accelerated apoptosis when they are exposed to multiple stimuli that normally induce DC maturation. Crucially, we also demonstrate that binding of gp120 to DC-SIGN results in excessive activation of p-ASK-1, and that silencing of ASK-1 effectively reverses this process and promotes DC survival.

## Results

### Cross-linked gp120 sensitizes cultured DC to undergo CD40L- and DC-SIGN-dependent apoptosis

**Figure 1:**
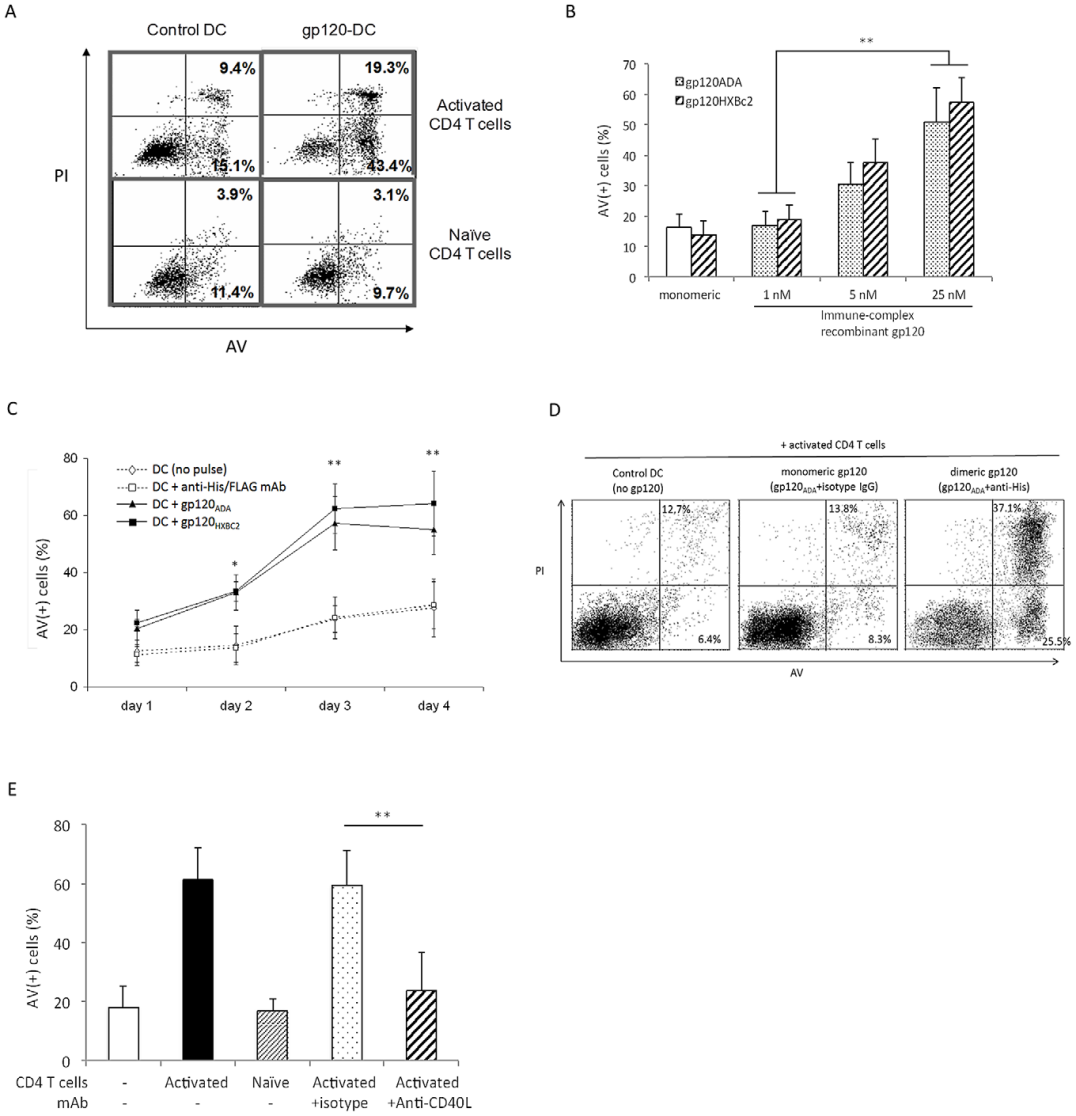
Cross-linked recombinant gp120 sensitizes moDC for CD40L-mediated apoptosis after co-culture with activated CD4 T cells. (***A***) moDC were treated for 24 h with anti-His mAb alone (control DC, left panels) or with 25 nM gp120_ADA_ cross-linked with anti-His mAb (gp120-DC, right panels), and co-cultured with autologous activated (upper panels) or naïve (lower panels) CD4 T cells for 3 d. The moDC (Fig. S1) were analyzed for Annexin V (AV) and propidium iodide (PI) expression to assess the extent of apoptosis, as manifested by the percentage of the AV-positive [AV(+)] cells. Data are representative of 5 experiments. (***B***) Apoptosis of moDC was analyzed after treatment with different concentrations of cross-linked recombinant gp120_ADA_ or gp120_HXBc2_ and co-culture with activated CD4 T cells for 3 d. DC treated with monomeric gp120 (not cross-linked with anti-His or anti-FLAG mAb) were used as a control. Data represent mean ± SD from 5 experiments; **p<0.01. The use of cross-linked recombinant gp120_BAL_ gave similar results (not shown). (***C***) Apoptosis of moDC was analyzed after treatment with the indicated cross-linked recombinant gp120, or appropriate mAb controls, at the indicated time points after co-culture with activated CD4 T cells. Data represent mean ± SD from 5 experiments (data for anti-His and anti-FLAG controls were indistinguishable); *P<0.05 and **P<0.01 compared with control group (DC with no gp120 pulse or DC plus anti-His/FLAG Ab). (***D***) moDC were respectively not treated (Control DC), or treated with gp120_ADA_ cross-linked with mouse IgG2a anti-His mAb (dimeric gp120-DC), or treated with cross-linked gp120_ADA_ supplemented with isotype control mouse IgG (gp120-DC+isotype IgG), and subsequently co-cultured with autologous activated CD4 T cells for 3 days before AV/PI staining. Data are representative of 3 experiments. (***E***) moDC were treated with cross-linked gp120_ADA_ and co-cultured for 3 d with autologous activated or naïve CD4 T cells, that had been pre-treated without or with 10 µg/ml isotype control or anti-CD40L mAb, before cell viability analysis. Data are expressed as mean ± SD from 5 experiments. **p<0.01.

We first studied the survival of gp120-pulsed DC that were exposed to activated CD4 T cells, mimicking the interactions that typically occur within secondary lymphoid tissues. Monocyte-derived DC (moDC) were treated with antibody cross-linked recombinant gp120 (gp120-DC) and co-cultured for 3 d with activated or naïve CD4 T cells. The gp120-DC were readily identifiable within these co-cultures as a distinct, large granular CD3(−)/DC-SIGN(+) cell population (Fig. S1). Analysis of Annexin-V (AV) and propidium iodide (PI) expression in this population revealed that a very high proportion of the gp120-DC underwent apoptosis following co-culture with activated, but not naïve, CD4 T cells (Fig. 1A, right upper *vs* lower panel). Treatment of moDC with cross-linked gp120 clearly induced apoptosis in both a dose-dependent (Fig. 1B) and time-dependent (Fig. 1C) manner. In our preliminary time-chase experiments, we followed AV/PI reactivity till d4 and found that the AV reactivity of DC reduced from d3 to d4, whereas that of PI increased over time, indicating that as incubation time increases, some cells at early apoptotic (AV+PI−) phase would become late apoptotic (AV+PI+) cells. Nevertheless, the overall AV(+) cells (*ie*, AV+PI− plus AV+PI+ cells) remained relatively consistent between d3 and d4 (Figure S1). We therefore chose d3 as our observation time point to compare the % of total AV(+) cells among various treatment conditions. Furthermore, while the cross-linked gp120 prompted DC for activated CD4 T cell-mediated apoptosis, treatment with monomeric gp120 did not (Fig. 1D). The formation of dimeric gp120 after cross-linking with anti-“tag” (anti-His or anti-FLAG Ab) has been confirmed by native non-reducing Western blot analysis (Fig. S2).

During the cross-talk between DC and activated CD4 T cells, CD40-CD40L interactions play crucial roles in both the regulation of DC survival and immune responses. To examine the potential involvement of CD40/CD40L interactions in the induction of apoptosis of gp120-DC, activated CD4 T cells were pre-treated with an antagonistic anti-CD40L mAb before co-culture with gp120-DC. Pre-treatment significantly inhibited the apoptosis of gp120-DC (Fig. 1E). Therefore, activated CD4 T cell-mediated apoptosis of immune-complex gp120-primed DC is at least in part CD40L-dependent.

### HIV-1 gp120 binding to DC-SIGN sensitizes moDC for apoptosis

**Figure 2:**
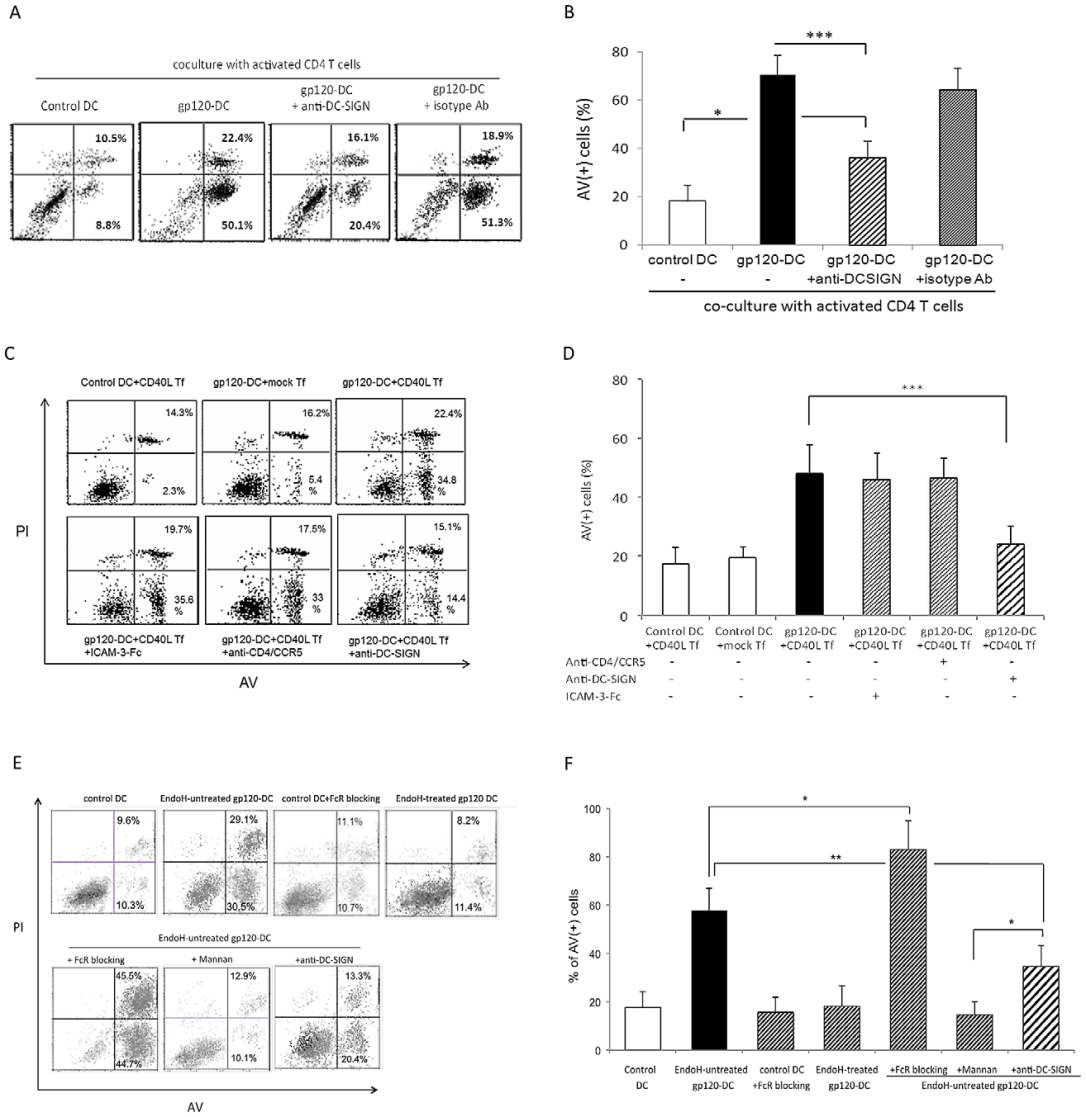
Cross-linked gp120 sensitizes DC through DC-SIGN and MCLRs for CD40L-mediated apoptosis. (***A***, ***B***) moDC were pretreated with anti-DC-SIGN mAbs or isotype control Ab before pulse with cross-linked gp120_ADA_ and co-culture for 3 d with autologous activated CD4 T cells, and subsequently subjected to cell viability assay. Data are representative of 3 experiments and are expressed as mean ± SD from 3 experiments in ***B***. (***C, D***) moDC were treated with cross-linked recombinant gp120_ADA_ with or without pre-treatment by soluble ICAM-3-Fc chimeric protein, anti-CD4 plus anti-CCR5 mAbs, or anti-DC-SIGN mAbs. Cells were subsequently co-cultured with mock- or CD40L-transfected (CD40L Tf) cells for 3 d. Data are representative of 7 experiments in panel ***C*** and are expressed as mean ± SD (n = 7) in ***D***; ***p<0.005. (***E, F***) Recombinant gp120_ADA_ were treated with or without EndoH, and then cross-linked with anti-His Ab before use to pulse moDC. Prior to gp120 pulsing, moDC were pre-treated with or without mannan or FcR blocking reagent, or anti-DC-SIGN mAbs for 30 minutes. After gp120 pulsing, DC were subsequently cocultured with CD40 Tf for 3 days. DC without any pre-treatment and only pulsed with anti-His Ab were used as a control (control DC). Data are representative of 3 experiments and expressed as mean ± SD from 3 experiments in ***F***; *p<0.05, **p<0.01.

As DC-SIGN is one of the major surface molecules for gp120 binding on moDC, we examined the role of DC-SIGN in the CD4 T cell-induced DC apoptosis by pre-treating DC with anti-DC-SIGN mAbs (a mixture of clones 120612 and DC28). The combination of anti-DC-SIGN mAbs used for these studies substantially reduced the binding of gp120 to DC-SIGN and inhibited HIV-1 uptake (Fig. S3) by the DC-SIGN-transfectants. Indeed, DC-SIGN blockade could significantly prevent DC apoptosis upon coculture with activated CD4 T cells (Fig. 2A & B). Because apoptosis of gp120-primed DC can be CD40L-dependent, we studied if exposure of gp120-DC to CD40L-transfected L (CD40L Tf) would lead to the same outcome. We analysed AV/PI expression of moDC (which expressed HLA-DR, Fig. S4) after separation from the adherent CD40L transfectants (which expressed high levels of CD40L and little HLA-DR, Fig. S4). We found that exposure to CD40L Tf cells also induced significant apoptosis of gp120-DC, compared with exposure to mock L cell transfectants (Fig. 2C, top panels, and Fig. 2D), and cell death was further confirmed by trypan blue staining; Fig. S*5*). We next tried to delineate the role of other gp120-binding receptors (apart from DC-SIGN) of moDC that might also be responsible for CD40L-induced apoptosis. We first pre-treated moDC with anti-CD4/chemokine receptor and anti-DC-SIGN mAbs, as previously described [38], and pulsed DC with cross-linked recombinant gp120 for coculture with CD40L Tf cells for 3 days. Results showed that in contrast to DC-SIGN blockade, pre-treatment with a combination of anti-CD4/chemokine receptor mAbs did not inhibit moDC apoptosis (Fig. 2C–D). Pre-treatment of moDC with cross-linked ICAM-3, a physiological ligand for DC-SIGN (Fig. S6) that does not prevent gp120 binding, was also ineffective in preventing apoptosis (Fig. 2C–D). Control studies demonstrated that pre-treatment with these antagonistic mAbs alone did not sensitize moDC for CD40-mediated cell death (Fig. S6). Taken together, we conclude that cross-linking of DC-SIGN, but not CD4/chemokine receptors, by recombinant gp120 sensitizes DC for apoptosis after CD40 ligation.

As HIV-1 gp120 binding to moDC has been shown to be exclusively carbohydrate-dependent [39], we also examined the role of carbohydrates of gp120 and if other mannose C-type lectin receptor (MCLRs), in addition to DC-SIGN, could also be involved in gp120/CD40L-mediated death. We adopted two approaches: removing the carbohydrate moieties of recombinant gp120 by EndoH (endo-β-N-glucosaminidase) before cross-linking with anti-“tag” Abs, and pretreating DC with mannan to compete off the gp120 binding. Because DC express abundant FcR [40] and FcR cross-linking may induce apoptosis of certain cells [41], [42], [43], we also investigated the effect of FcR blockade prior to gp120 pulsing. After EndoH treatment, the deglycosylated monomeric gp120 had reduced molecular weight (≈80 kDa, reduced from ≈120 kDa), and after cross-linking in dimeric form, they indeed lost their binding capacity to moDC (Fig. S2). Furthermore, the immune-complex EndoH-treated gp120 lost the ability in sensitizing DC for apoptosis upon CD40 ligation (Fig. 2E, upper row, and Fig. 2F). Pre-treatment of moDC with mannan prior to gp120 pulsing also completely prevented the CD40L-mediated apoptosis, which was more potent than mere DC-SIGN blockade. Moreover, while FcR blockade itself did not induce CD40L-mediated apoptosis of control DC (pulsed with anti-“tag” Ab), it significantly promoted further the extent of CD40L-mediated apoptosis of gp120-DC (Fig. 2E, lower row, and Fig. 2F).

### Immune complexes of gp120 from the sera of HIV-positive individuals can sensitise cultured dendritic cells for apoptosis

**Figure 3:**
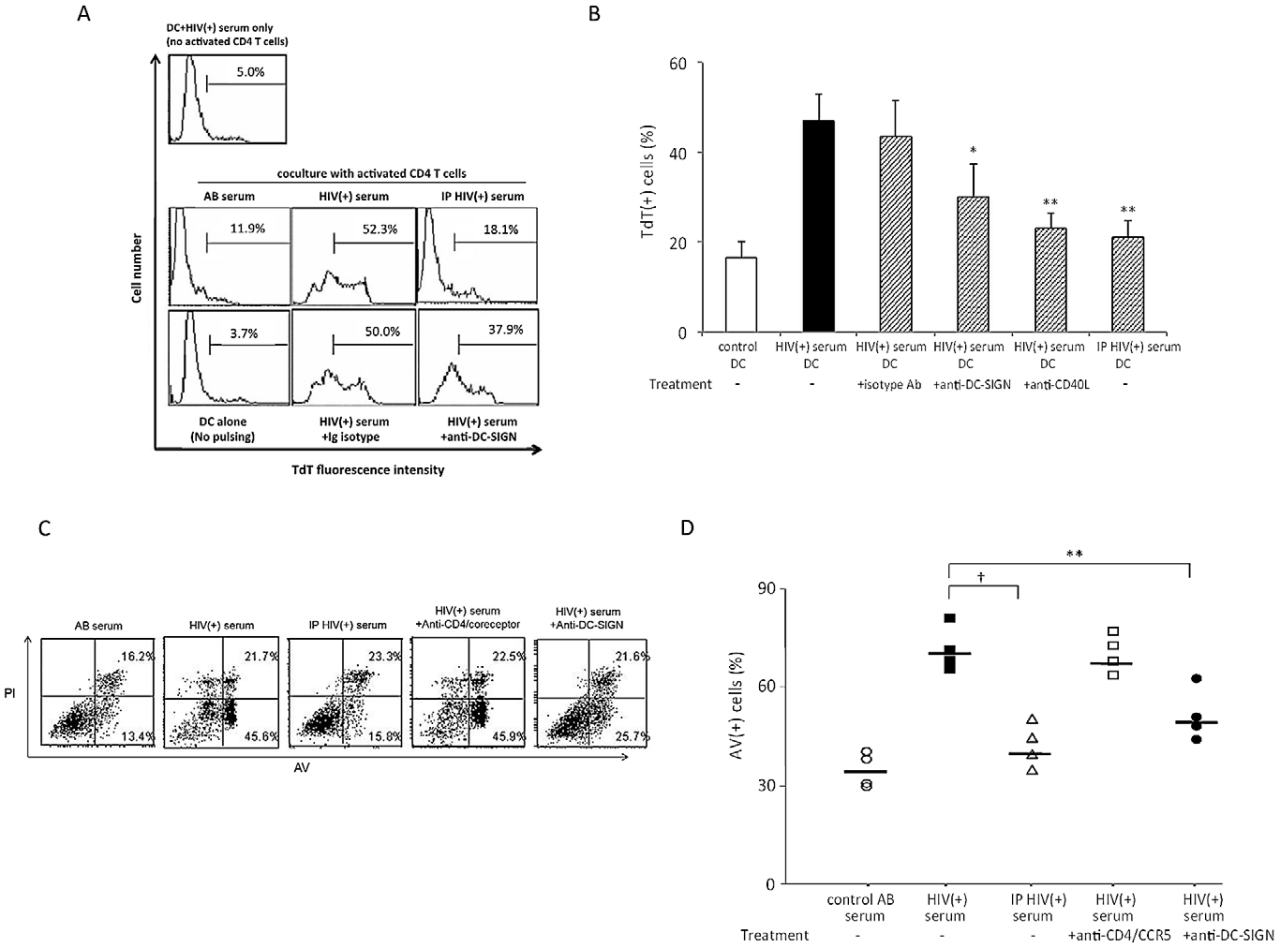
Sera from HIV-1(+) individuals can sensitize moDC for DC-SIGN dependent CD40L-mediated apoptosis. (***A***) moDC were treated with HIV(+) serum before or after immunoprecipitation (IP) with anti-gp120 mAbs, or with or without anti-DC-SIGN or isotype control mAbs, and subsequently co-cultured with autologous activated CD4 T cells. After 3 d, cells were harvested and subjected to TUNEL assays. Cell death was assessed as the percentage of cells expressing terminal deoxynucleotidyl transferase (TdT). DC pulsed with HIV(+) serum without coculture with activated CD4 T cells (top panel) were also used as a control. Data are representative of 4 experiments. (***B***) MoDCs were treated with anti-DC-SIGN mAbs, isotype control Ab, or anti-CD40L mAb before pulse with HIV serum (before or after immunoprecipitation of gp120) and cocultured with activated CD4 T cells. Data are expressed as mean ± SD (n = 4); *p<0.05 and **p<0.01 compared with ‘HIV(+) serum-DC plus isotype Ab’. (***C***,***D***) moDC were treated with normal AB serum or HIV-1(+) serum with viral RNA copies >400,000/ml (Table S1), with or without pre-treatment with anti-CD4 plus chemokine receptor or anti-DC-SIGN mAbs, or with gp120-depleted (immunoprecipitated, IP) HIV(+) serum, and co-cultured with CD40L Tf for 3 d. Data are representative of 4 experiments in panel ***C*** and individual datum with the mean is shown in panel ***D***; †: P<0.001 between with and without IP of gp120 from the HIV serum, **: P<0.01 between with and without pre-treatment with anti-DC-SIGN mAbs.

To investigate whether sera from HIV-1-positive (+) individuals can contain sufficient levels of gp120 immune complexes to sensitize moDC for CD40L-mediated apoptosis, we next treated moDC with HIV-1(+) sera containing high viral copy numbers (>400,000/ml) (Table S1). In order to eliminate the effect of other soluble immune factors, the sera were first centrifuged through filters with a cut-off point of 100 kDa and the >100 kDa fractions were collected and reconstituted to the original volume with fresh medium before use, as described [27]. After 3 d co-culture with autologous activated CD4 T cells, the HIV-1(+) sera-treated moDC were identified as a smaller-sized population than the control moDC, and they were CD3(−) (Fig. S7). These cells were then subjected to TUNEL assays with flow cytometric analysis to evaluate terminal deoxynucleotidyl transferase (TdT) expression as a measure of apoptosis. This confirmed that HIV(+) serum-pulsed moDC had undergone remarkable apoptosis (∼50%, Fig. 3A), and that this could be substantially inhibited by pre-treatment with anti-DC-SIGN mAbs (Fig. 3A–B) or anti-CD40L mAbs (Fig. 3B). Furthermore, excessive apoptosis of moDC was effectively prevented after removal of gp120 from the HIV-1(+) sera by immunoprecipitation (IP) (Fig. 3A–B), under conditions that also depleted virions from cultures of live virus (Fig. S8). Similar results were obtained following co-culture of HIV-1(+) serum-treated moDC with CD40L Tf cells, and further confirmed that CD4 and chemokine receptors were not involved in sensitization for apoptosis (Fig. 3C–D). Therefore, HIV-1(+) sera may contain sufficient levels of immune complexed and/or virion-bound gp120 to sensitize moDC for CD40/CD40L-mediated apoptosis.

**Figure 4:**
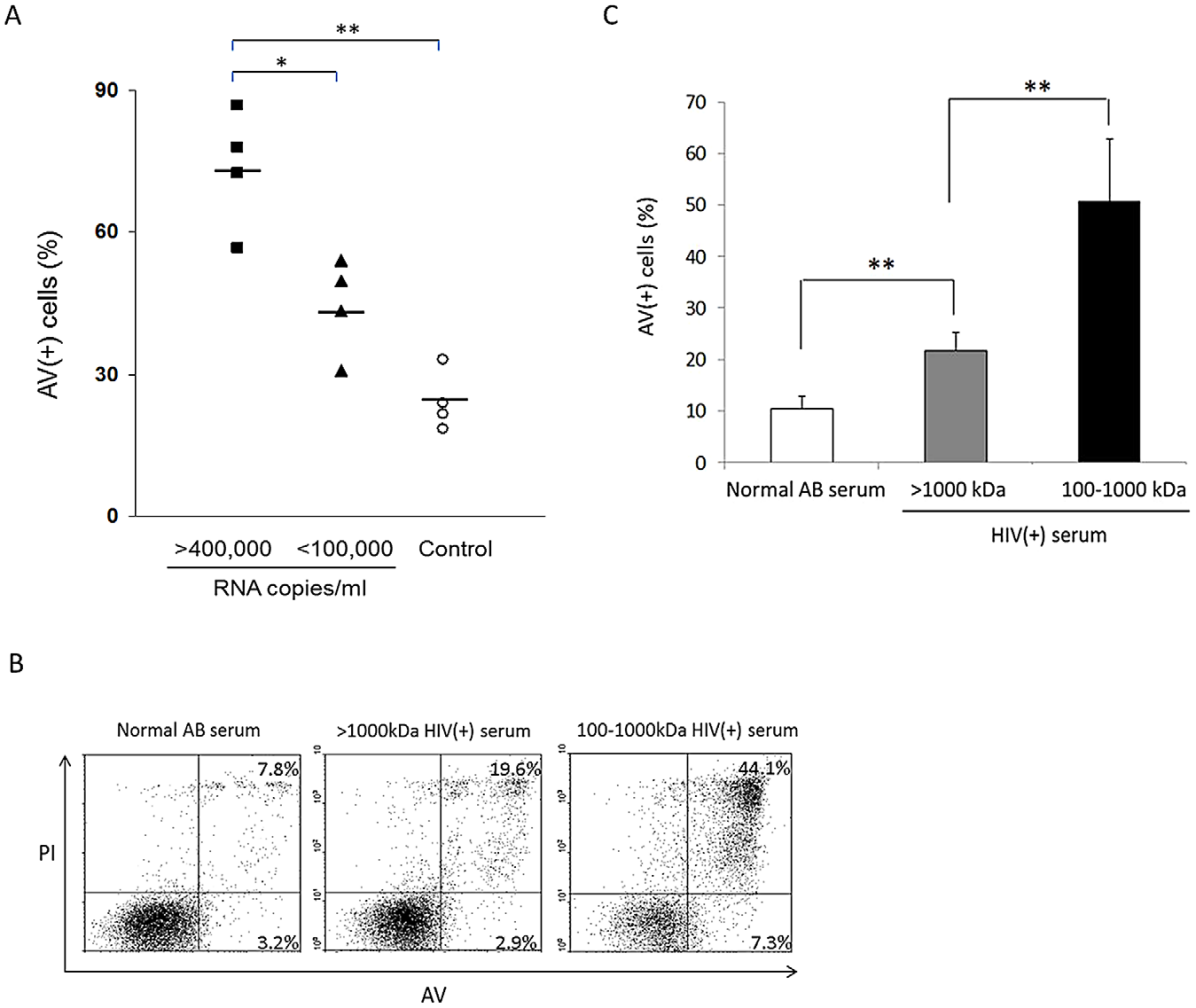
CD40L-mediated apoptosis of HIV(+) serum-pulsed DC is proportional to viral loads and predominantly induced by the 100–1000 kDa fraction. (***A***) moDC were treated with HIV-1(+) sera with viral copy numbers >400,000/ml or <100,000/ml or normal AB serum (control) and co-cultured with CD40L Tf cells for 3 d. Data are expressed as individual datum with the mean. N = 4 for each condition; *p<0.05 and **p<0.01. (***B, C***) moDC were treated for 24 h with normal AB serum or with >1000 kDa or 100–1000 kDa fractions from HIV(+) serum and were co-cultured with CD40L Tf for 3 d. Data are representative of 5 experiments in ***B*** and expressed as mean ± SD in ***C***; ** p<0.01.

We next investigated the relative contributions of circulating gp120 and virions from HIV(+) serum in sensitizing DC for apoptosis. First, we treated moDC with >100 kDa fractions of sera containing either relatively low (<100,000/ml) or high (>400,000/ml) viral copy numbers (Table S1). After co-culture with CD40L Tf cells, the extent of DC apoptosis was significantly higher in treatment with serum of relatively high HIV RNA viral loads (Fig. 4A). We next further fractionated the >100 kDa serum fractions into virion-free (100–1000 kDa) and virion-enriched (>1000 kDa) portions. The moDC were then treated with these respective fractions and co-cultured with CD40L Tf cells. We detected slightly higher levels of apoptosis after treatment with the virion-enriched portions than controls, but substantial levels of apoptosis was observed after treatment with the virion-free (gp120-enriched) portion (Fig. 4B–C). Hence gp120, circulating as immune complexes in HIV(+) sera, plays a more significant role than virus-bound gp120 in sensitizing DC for CD40/CD40L-dependent apoptosis.

In separate experiments, we also examined the sensitizing capacity of EndoH-treated gp120, pre-treatment with mannan, as well as FcR blockade of DC for CD40L-mediated apoptosis. Consistently as in the case of recombinant gp120 (Fig. 2E & F), EndoH-treated HIV(+) serum had lost the capacity in priming DC for CD40L-mediated apoptosis. Furthermore, compared with DC-SIGN blockade, pre-treatment of DC with mannan (before pulse by HIV(+) serum) further prevented DC apoptosis (down to background levels). FcR blockade of DC prior to exposure to HIV serum further increased the extent of DC death (Fig. S9).

### Blood DC from healthy individuals can be sensitized for apoptosis by gp120, and are pre-sensitized within the circulation of HIV-1-positive individuals

**Figure 5:**
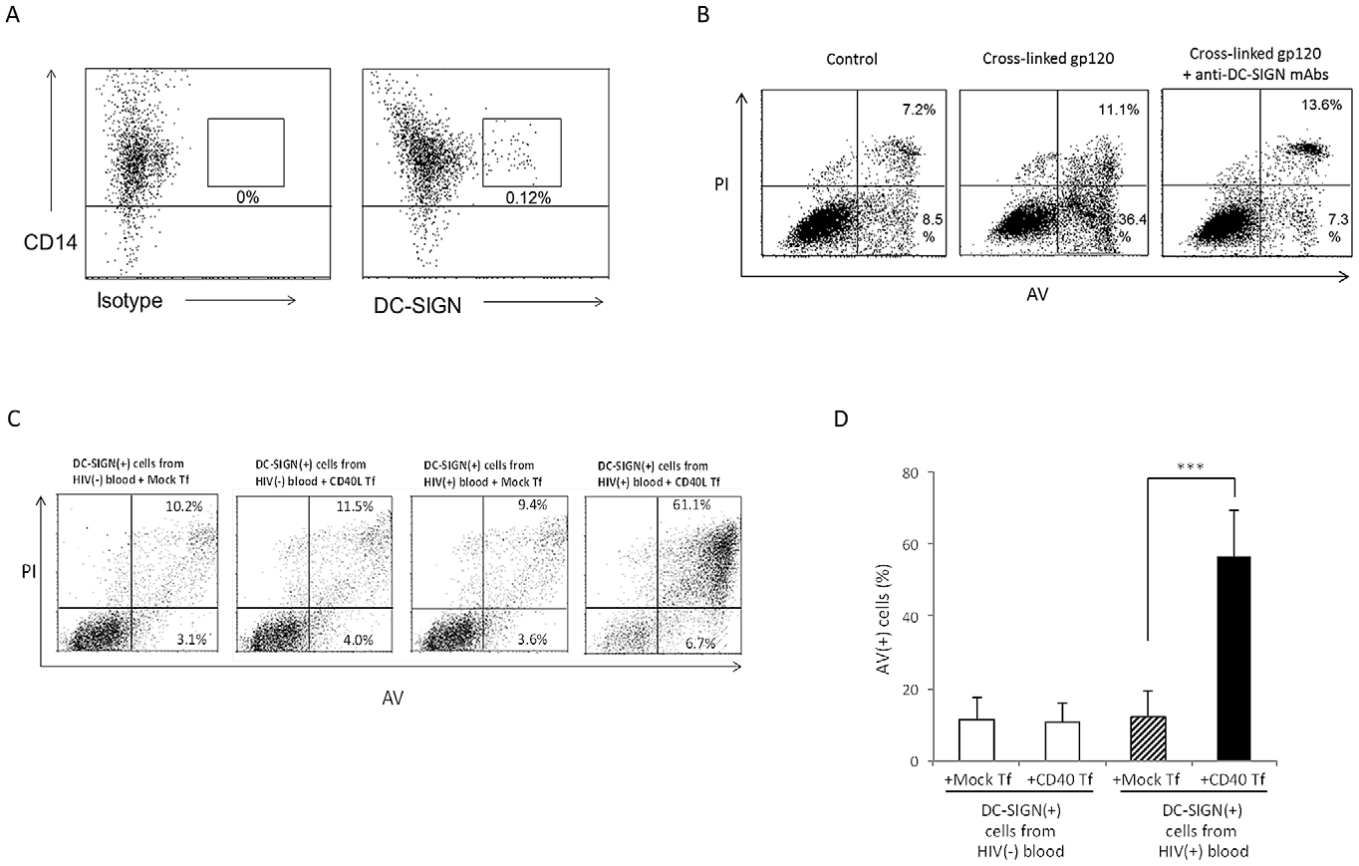
Freshly-isolated DC-SIGN(+) blood DC underwent DC-SIGN-dependent CD40L-mediated apoptosis and DC-SIGN(+) cells from HIV-1-infected individuals are pre-sensitized for CD40L-mediated apoptosis. (***A***) PBMCs from normal HIV(−) individuals were labelled with anti-CD14 plus either isotype control (left panel) or anti-DC-SIGN (right panel) mAbs and analysed by flow cytometry for cell isolation. Data are representative of 4 experiments. (***B***) Purified CD14(+)DC-SIGN(+) cells were treated with anti-His mAb alone (Control) or anti-His cross-linked recombinant gp120_ADA_, in the absence or presence of anti-DC-SIGN mAbs, and subsequently co-cultured with CD40L Tf for 3 d. The non-adherent DC were then harvested and subjected to cell viability assay. Data are representative of 4 experiments. (***C, D***) freshly isolated DC-SIGN(+) cells from HIV(+) and HIV(−) blood were cocultured with mock Tf or CD40L Tf for 3 days and subjected to cell viability assay. Data are representative of 4 experiments in ***C*** and expressed as mean ± SD in ***D***. ***p<0.005.

To extend our studies beyond the use of *in vitro*-generated DC, we next purified the DC-SIGN(+) subset of DC from peripheral blood of normal individuals. Approximately 0.12–0.3% of CD14(+) cells in blood expressed DC-SIGN (Fig. 5A; equivalent to 0.01–0.03% of PBMCs), which also expressed CD40 and CD11c (Fig. S10), consistent with a previous report [9]. After priming by cross-linked gp120 and coculture with CD40L Tf cells, these blood-derived DC also underwent excessive apoptosis, and this was significantly reduced by prior blockade of DC-SIGN (Fig. 5B). Hence this *bona fide* primary human blood DC subset can be sensitized through gp120 ligation of DC-SIGN for CD40/CD40L-dependent apoptosis.

Based on these and other findings above, we then hypothesized that DC-SIGN(+) DC in the peripheral blood of HIV-1(+) individuals would be excessively vulnerable to apoptosis due to their continual exposure to high levels of immune-complex gp120 in the circulation. We therefore also isolated the DC-SIGN(+) DC subset directly from the blood of HIV-1(+) individuals and found that a substantial degree of apoptosis occurred after they were cultured with CD40L Tf cells, whereas the same subset of DC from HIV(−) individuals did not (Fig. 5C–D). We thus conclude that DC-SIGN(+) DC from healthy human blood can be sensitized for apoptosis after *in vitro* exposure to cross-linked gp120, and DC-SIGN(+) in the circulation of HIV(+) individuals may have been pre-sensitised *in vivo* by circulating immune complexes of gp120.

### HIV-1 gp120 sensitizes cultured DC and blood DC for apoptosis after exposure to LPS, TNF-α and IL-1β

**Figure 6:**
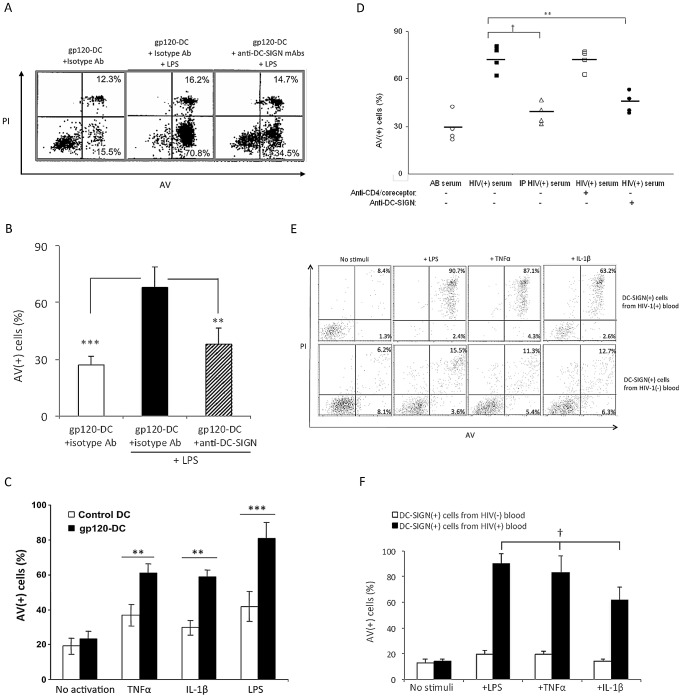
Cross-linked recombinant gp120 or HIV(+) serum sensitizes moDC for apoptosis after activation by LPS, TNF-α or IL-1β, and DC-SIGN(+) cells in HIV(+) blood are pre-sensitized for LPS/TNFα/IL-1β-induced apoptosis. (***A,B***) moDC were treated with gp120_ADA_ in the presence of isotype control or anti-DC-SIGN Abs and subsequently cultured in the absence or presence of 100 ng/ml LPS for 3 d. Data are representative of 5 experiments in ***A*** and are expressed as mean ± SD in ***B***; **p<0.01, ***P<0.005. Similar findings were made after treatment with gp120_BAL_ or gp120_HXBc2_ (not shown). (***C***) moDC were treated with anti-His cross-linked recombinant gp120_ADA_ (gp120-DC) or anti-His Ab alone (Control DC), and cultured for 3 d in the absence (no activation) or presence of 100 ng/ml LPS, TNF-α or IL-1β. Data represent mean ± SD from 4 experiments; **p<0.01 and ***p<0.005. (***D***) moDC were treated with normal AB serum or HIV-1(+) sera (viral RNA copies >400,000/ml; n = 4) before or after gp120 immunoprecipitation (IP), with or without pre-pretreatment with anti-CD4 plus anti-CCR5, or anti-DC-SIGN mAbs and subsequently exposed to 100 ng/ml LPS for 3 d; **P<0.01 and †P<0.001. (***E,F***) DC-SIGN(+) cells from HIV(+) and HIV(−) blood were cultured in the absence (‘No stimuli’) or presence of 100 ng/ml of LPS, TNFα, or IL-1β for 3 d prior to analysis of cell viability. Data are representative of 4 experiments in ***E*** and expressed as mean ± SD (n = 4) in ***F***. †P<0.001 between cells from HIV(+) blood and from HIV(−) blood.

We next studied the survival of gp120-primed DC after exposure to typical stimuli that might be encountered at peripheral sites of infection and inflammation, prior to DC migration to lymphoid tissues. MoDC were treated with cross-linked recombinant gp120 and subsequently cultured with 100 ng/ml LPS, TNF-α or IL-1β for 3 d. Very considerable levels of apoptosis were induced in gp120-primed DC after exposure to LPS, and this could be significantly prevented by prior blockade of DC-SIGN (Fig. 6A–B). Likewise, substantial levels of apoptosis were also induced after exposure to TNF-α and IL-1β (Fig. 6C). Excessive apoptosis was further induced when the cross-linked gp120 was replaced by HIV-1(+) sera containing high viral loads (>400,000 RNA copies/ml) prior to LPS stimulation, but was prevented by prior immunoprecipitation of gp120 from the sera and substantially reduced by blockade of DC-SIGN, but not CD4/CCR5 (Fig. 6D).

To extrapolate our findings to the *in vivo* setting, DC-SIGN(+) cells from the peripheral blood of HIV-1(+) or HIV-1(−) individuals were isolated and exposed to 100 ng/ml LPS, TNFα or IL-1β. While there was little apoptosis of blood DC from HIV(−) individuals, blood DC-SIGN(+) DC from HIV(+) individuals underwent substantial apoptosis, indicating that they are pre-sensitized *in vivo* to die after contact with multiple stimuli that otherwise induce DC maturation (Fig. 6E–F).

### Binding of HIV-1 gp120 to DC-SIGN promotes ASK-1-dependent apoptosis of human dendritic cells

To explore potential mechanisms by which gp120 ligation of DC-SIGN sensitizes DC for apoptosis, we first noted that treatment of moDC with cross-linked gp120 alone was able to modulate the expression of key membrane molecules in a manner that is typically associated with DC maturation (Fig. S11), as reported by others [44]. Together with our earlier observations (Fig. 1C; *cf.* apoptosis of gp120 at d2 *vs.* control DC at d4), this finding suggested that gp120 ligation of DC-SIGN greatly accelerates an apoptotic programme that is normally induced following maturation.

**Figure 7:**
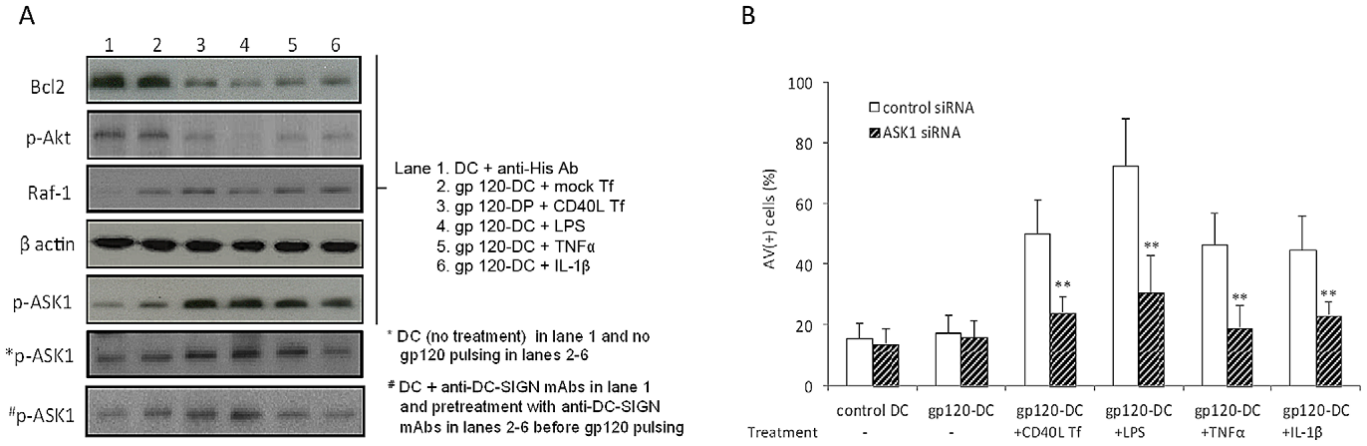
Activation of gp120-primed DC reduced the expression of Bcl2 and activated Akt, and the induction of cell apoptosis is ASK1-dependent. (***A***) moDC treated with immune-complex gp120_ADA_ were exposed to CL40L Tf or mock Tf, or LPS, TNFα, IL-1β for 3 d, and cellular proteins of moDC (recovered from coculture) were extracted for Western blotting analysis. MoDC treated by anti-His Ab (used to cross-link recombinant gp120_ADA_) were used as a control (control DC). *p-ASK1 represent results from untreated moDC (lane 1) and DC treated as indicated but no gp120 pulsing (lanes 2–6), whereas #p-ASK-1 represents results from pre-treatment of anti-DC-SIGN mAbs. Data are representative of 3 experiments. (***B***) moDC were transfected with siRNA against human ASK1 or control siRNA (scrambled) before pulse with immune-complex gp120 and subsequent exposure to CD40L Tf, LPS, TNFα or IL-1β. Data are expressed as mean ± SD from 3 experiments. **p<0.01.

We then studied components of the intracellular signalling pathways that might be involved in gp120 sensitization of DC for apoptosis. MoDC were treated with cross-linked gp120, or with HIV(+) sera, and co-cultured with CD40L Tf cells or exposed to LPS, TNFα or IL1β (as above). After 3 days, moDC were harvested from the cultures and Western blot assays were performed to analyze the expression of key molecules regulating the balance of DC survival *versus* death. First, we noted a substantial reduction in the expression levels of the anti-apoptotic molecules Bcl-2 (Fig. 7A), and Bcl-xL (Fig. S12), after exposure of cross-linked gp120-DC (Fig. 7A) or HIV(+) sera-treated moDC (Fig. S12) to the above stimuli. Second, turning to upstream components that may lead to these changes we found that p-Raf-1, which can be activated by the DC-SIGN signalosome after gp120 binding, was increased as expected but little further change occurred after exposure to the various stimuli. In marked contrast, each of these stimuli resulted in a marked reduction of p-Akt which is anti-apoptotic, but a very substantial increase in p-ASK1 which is pro-apoptotic. Studying the latter in more detail, we found the expression of p-ASK1 could be reduced virtually to baseline levels (moDC with stimuli alone) by DC-SIGN blockade (Fig. 7A, lower three panels).

The above observation indicates that gp120 ligation of DC-SIGN, either directly or indirectly, leads to excessive ASK1 activation which promotes DC apoptosis. To confirm the role of ASK1, we transfected moDC with ASK1-specific siRNA before treatment with gp120 and exposure to different activation stimuli. In all cases, silencing of ASK1 substantially prevented excessive DC apoptosis in response to each of the activation factors (Fig. 7B). Therefore, gp120 ligation of DC-SIGN accelerates an apoptotic programme that normally accompanies DC maturation, and which involves excessive ASK-1 activation that results in unusually premature cell death.

## Discussion

In chronically-infected HIV-1(+) individuals, multiple subsets of DC in peripheral blood [29]–[31], [36] and lymphoid tissues [12], [34] become progressively depleted during disease progression to AIDS, and the reduction of DC number is proportional to the viral RNA loads [29], [35], [37]. It is unlikely that direct infection and killing of DC by HIV-1 can account for this substantial depletion, given the remarkably low frequency of cells that are actually infected. We present in this study a gp120-exerted mechanism that may account, at least in part, for the progressive depletion of conventional DC subsets, as seen in chronic HIV infection. We provide evidence that moDC can be sensitized by cross-linked recombinant gp120 (Fig. 1 and 2), and HIV-1(+) sera (Fig. 3 and 4), to undergo greatly accelerated apoptosis in response to CD40 ligation, which typically occurs after DC migration into lymphoid tissue. The extent of DC apoptosis was proportional to the concentration of recombinant gp120 (Fig. 1B) and viral RNA loads in the sera (Fig. 4A). These observations were reinforced by the finding that CD14(+)DC-SIGN(+) DC, isolated directly from the blood of healthy [HIV(−)] individuals, could also be sensitized by gp120 for CD40L-induced cell death (Fig. 5). Immunoprecipitation of gp120 from HIV-1(+) sera substantially reduced the extent of apoptosis, showing that either virion-bound gp120 and/or gp120 in the circulating immune-complex form could sensitize the cells (Fig. 3). When we further fractionated the HIV(+) sera, we found that the 100–1000 kDa (virion-free) portion promoted substantial levels of apoptosis after CD40 ligation, whereas considerably lower levels were induced by the >1000 kDa (virion-enriched) portion (Fig. 4B–C). Since the molecular weight of IgG is ∼150–170 kDa, circulating immune-complex gp120 would be contained in the virion-free fractions (100–1000 kDa). Therefore, gp120 in immune complexes is primarily responsible for sensitizing DC for apoptosis, and HIV(+) serum we tested contains sufficient quantities of circulating gp120 *in vivo* to sensitize DC-SIGN(+) DC for apoptosis. However, it remains to be studied if the smaller amount of sensitization by the virion-rich fraction might be induced by activities other than mere gp120/DC-SIGN binding (*e.g.*, events after viral entry). To evaluate our findings further and more directly, we isolated CD14(+)DC-SIGN(+) DC from the blood of HIV(+) individuals and found that they were indeed ‘pre-sensitized’ for apoptosis on CD40 ligation (Fig. 5). Moreover, both these blood DC from HIV(+) individuals and moDC sensitized by recombinant or serum gp120 underwent substantial apoptosis after exposure to bacterial LPS and the pro-inflammatory cytokines TNF-α and IL-1β (Fig. 6), apart from CD40 ligation.

Amongst HIV-1 encoded proteins, the envelope protein gp120 has two very unusual properties, even for a retroviral envelope component: first, gp120 is only loosely attached to the virion and is rapidly shed in large quantities into the circulation [3], and second, liberated gp120 binds to and activates a very high proportion of B cells [28], [45], resulting in a massive overproduction of anti-gp120 antibodies and the formation of increasing amounts of immune complexes in the serum. Our findings now reveal that through ligation of immune-complex gp120 to DC-SIGN and mannose C-type lectin receptors (MCLRs), apoptosis of DC is remarkably promoted when the cells encounter diverse maturation stimuli that are otherwise prerequisites for the effective initiation and regulation of many immune responses. The concentration of “free gp120” in the HIV(+) serum has been studied by many groups, varying from “pM” to “nM” levels [3], [4], [46], [47]. However, most reports determining the free gp120 in HIV(+) serum employed an antigen capture assay (*eg*, ELISA) without taking into account the presence of anti-gp120 Ab in the serum, which could significantly mask the detection of free gp120 and sometimes completely abrogate the signals [4]. The levels of “free gp120” detected in previous reports, thus, may not represent those of antibody-bound gp120. It has been demonstrated that anti-gp120 Abs are present in the HIV(+) serum at high enough concentrations to bind most of gp120 [48] and the levels of anti-gp120 Abs have been estimated to be in the micromolar range [49]. Therefore, for high affinity binding (*K*d<10 nM between gp120 and anti-gp120 Ab), virtually all the gp120 in the HIV(+) serum might have been occupied (saturated) by antibodies [48]. In a recent report, it has been estimated that the gp120 levels could be up to 5 µg/ml when concentrations of all forms (soluble and cell/virion-bound) are added, in situations of high plasma viremia [47]. The concentration of the immune-complex gp120 we used in the current study, therefore, is not incompatible with the *in vivo* scenario. Furthermore, it is interesting to note that even in the same experimental model, DC might have different reactivity over AV or PI in the viability assay. Throughout the entire study, around 50% of the donors had more exaggerated PI staining, while the other had more prominent AV staining (for instance, Fig. 5B *vs* 5C). It is likely that DC from different individuals may have different responsiveness to activation signals such as CD40 ligation, a phenomenon of “donor-to-donor variation”, which has been recently reported for HIV gp120 binding to moDC to induce IL-10 and impair IL-12 production [24]. Differential responsiveness to CD40 ligation in DC has also been reported that in mouse DC which express CD40^high^ could produce more IL-12 but less IL-10 than those produced by the CD40^low^ DC [50]. It deserves further investigation to see if the expression levels of CD40 of moDC correlate with the degree of CD40L-mediated apoptosis, and if DC apoptosis may also be accompanied by IL-10 production [24].

In studies to identify the membrane receptor(s) on DC through which cross-linked gp120 and HIV(+) sera can sensitize the cells, we observed that apoptosis was considerably reduced after pre-treatment with antagonistic mAbs specific for DC-SIGN, but not a combination of CD4 and chemokine receptors CCR5 or CXCR4 (Fig. 2A–D, Fig. 3C–D, 5B and 6D). In contrast, pre-treatment of DC with a cross-linked, Fc construct of ICAM-3, a physiological ligand for DC-SIGN important for stabilizing the DC-naïve T cell immune synapse [51], was ineffective (Fig. 2C–D). This is in accordance with previous reports that the binding site for ICAM-3 is distinct from that of gp120 [52], and that soluble ICAM-3 is not able to compete efficiently with HIV-1 envelope protein or with intact viruses for binding to DC-SIGN [53]. Notably, the inhibition of apoptosis by DC-SIGN blockade was substantial but incomplete, apparently reflecting the relative capacity of the combination of mAbs to prevent binding of gp120 to DC-SIGN (Fig. S3). Furthermore, other MCLRs in addition to DC-SIGN that can bind gp120 [32] are also involved in DC apoptosis, because pre-treatment of DC with mannan led to virtually complete diminishment of the CD40L-mediated DC death, in contrast to partial (albeit significant) inhibition by DC-SIGN blockade (Fig. 2E–F). Moreover, we agree with a previous report [39] and confirm that gp120 binding to moDC is exclusively carbohydrate-dependent because the binding capacity of deglycosylated gp120 (whose carbohydrate moieties were removed by EndoH treatment) to moDC was completely lost (Fig. S2), resulting in total abrogation of CD40L-mediated apoptosis of gp120-DC (Fig. 2E & F). Similar effects were also demonstrated in the use of HIV(+) serum (Fig. S9).

We also noted that sensitization of DC for apoptosis was only achieved with cross-linked recombinant gp120, and presumably gp120 immune complexes in HIV(+) sera, but not with gp120 in monomeric form (Fig. 1E). This suggests that cross-linking of DC-SIGN is required for sensitization. Indeed, antibody cross-linking of DC-SIGN [54] and the binding of HIV-1 gp120 [8] or many other pathogens [55]–[58] have been shown to initiate diverse intracellular signalling pathways in DC that lead to different cellular responses. Along with this thought, we blocked the Fc receptor (FcR) of moDC (before pulse by immune-complex gp120 or HIV(+) serum) in order to allow more potent clusterization of DC-SIGN or other MCLRs. This indeed resulted in more abundant DC apoptosis (Fig. 2E–F & Fig. S9). This finding further supports the involvement of DC-SIGN or MCLRs in gp120 sensitization for CD40L-induced DC death. Another point to support DC-SIGN for gp120-triggered intracellular signalling is that, apart from sensitizing DC for accelerated activation-induced death, gp120 can also induce phenotypic changes of DC that are typical of maturation (Fig. S11). The role of MCLRs in gp120-mediated DC phenotypic maturation has also been reported by others [44]. Therefore, immune-complex gp120 binding to DC-SIGN (or other MLCRs) may have sensitized DC into a “pre-mature” state, which may be a pre-requisite that prompted DC to be more vulnerable to activation-induced apoptosis. However, when compared with conventional maturation (*e.g.*, LPS/TNFα/IL-1β and CD40 ligation), the gp120-induced phenotypic “maturation” appeared inefficient, as manifested by less remarkable upregulation of CD80, CD86, CD83 and CCR7. The CCR5 downregulation was also less noticeable than that induced by conventional factors; in contrast, MHC class II upregulation remained relatively unaffected (Fig. S11). Such inefficient maturation is in line with a previous report that gp120 binding to MCLRs can induce immunosuppressive responses from DC, such as IL-10 production, which may subsequently contribute to a weaker T cell stimulation capacity [24].

In exploring the mechanistic basis for the gp120 sensitization process, we observed marked decrease in the downstream anti-apoptotic components Bcl-2 (Fig. 7A) and Bcl-xL (Fig. S12). Turning to upstream components that may account for these observations and explain excessive apoptosis, we found that gp120 binding activated Raf-1, an anti-apoptotic through its activity to restrict caspase activation [41], as reported by others [22], and its expression was not significantly modulated upon exposure to maturation stimuli (Fig. 7A). Strikingly, however, anti-apoptotic p-Akt was markedly reduced whereas pro-apoptotic ASK-1 was very substantially increased. Crucially, pre-treatment of gp120-DC with anti-DC-SIGN mAbs reduced ASK-1 activation back to control levels (Fig. 7A), while siRNA silencing of this component substantially prevented DC apoptosis (Fig. 7B). Hence, gp120 binding to DC-SIGN activates ASK-1 and further exposure to the different maturation stimuli results in abundant p-ASK-1 expression which may account for the excessive levels of apoptosis. Whether or not this is directly due to activation of a novel ASK-1-dependent signalling pathway after gp120 ligation of DC-SIGN, or another pathway that releases inhibition by p-Akt, deserves further attention. Furthermore, since HIV-1 Nef can suppress ASK-1 activation in infected T cells to protect them from apoptosis [49], it would be interesting to determine whether or not infected DC can be protected in a similar manner while the uninfected ‘bystander’ cells are killed.

Based on our findings, we propose that the capacity of gp120 to ligate DC-SIGN and sensitize conventional DC for apoptosis after encounter with diverse activation stimuli that otherwise induce cellular maturation may account for the depletion of DC-SIGN(+) DC, which has been clearly demonstrated in lymph nodes of HIV(+) patients [12] and in spleens of SIV-infected non-human primates [12]. We further propose that during disease progression to AIDS, this may contribute to the decreased capacity to mount effective immune responses [15]–[17], [59] and also to increased susceptibility to multiple opportunistic infections. In fact, the types of infection that are seen during AIDS progression somewhat resemble those described for some of the recently identified DC deficiency syndromes [13]. The presence of LPS, resulting from, for example, the mucosal barrier of the GALT being compromised or concomitant bacterial infections [14], [50], can also potentiate HIV-induced immunosuppression through augmentation of HIV-1 gene expression at least in part by stimulating the secretion of TNF-α and IL-1β [60]; high levels of circulating TNF-α can also be detected in HIV-infected individuals [61]. These factors could well predispose ‘bystander’ DC-SIGN(+) cells, which have been pre-sensitized by gp120 in the serum, for exorbitant apoptosis in the lymphoid tissues or blood.

In a broader context, it is clear that many other pathogens may also bind DC-SIGN to subvert DC functions, and DC-SIGN ligation can modulate TLR-associated activation [55]–[58]. It therefore deserves further study if such pathogens can also decrease the survival of DC through their capacity to cross-link DC-SIGN. Also, whether the subsequent modulation of TLR responsiveness can additionally or synergistically contribute to the defects of pathogen-specific immunity in AIDS warrants further investigation. In a narrower context, it is also clear that many other mechanisms can contribute to depletion of different subsets of DC during HIV-1 infection, including negative control of the numbers of plasmacytoid DC by type I interferons [28]. Whether or not binding of gp120 to other membrane receptors of DC can sensitize the respective subsets for apoptosis also needs to be further clarified. Finally, accelerated activation-induced apoptosis of gp120-sensitized DC is accompanied by excessive activation of ASK-1, and that silencing of ASK1 prevents apoptosis, implying that antagonistic ASK1 therapies, such as have been shown to reduce SIV encephalitis in macaques [48], might be of value to prevent DC depletion during HIV-1 disease progression.

## Materials and Methods

### Preparation of monocyte-derived DC and treatment with recombinant gp120 treatment (gp120-DC)

Monocyte-derived DC (moDC) were generated by culture for 6–7 d in RPMI1640 supplemented with 5% autologous serum, GM-CSF and IL-4 as described [27]. The moDC were then purified by two rounds of immunomagnetic depletion (Dynabeads, Dynal, Oslo, Norway) using monoclonal antibodies (mAbs) against CD3, CD8, CD14, CD16, CD19, and CD56 (BD PharMingen, San Diego, CA, USA); the resultant HLA-DR^high^ cell population was >98% pure (data not shown). Recombinant His-tagged gp120_ADA_, and FLAG-tagged gp120_BAL_ and gp120_HXBc2_, were prepared as described [27]; each contained <5 EU/ml LPS by Limulus amebocyte lysate (LAL) assay. 1×10^5^ moDC were cultured in 6-well tissue culture plates (Corning Life Sciences Corp., USA) at 37°C for 24 h in culture medium as described [27] with 50 nM recombinant R5 (HIV-1_ADA_ and HIV-1_BAL_), or X4 (HIV-1_HXBc2_) gp120. Before use, the gp120 was cross-linked by incubating for 24 h at 37°C with respective anti-His (mouse IgG2a isotype) or anti-FLAG (mouse IgG1 isotype) mAbs (Sigma–Aldrich, USA) at a molar ratio 2∶1 (gp120 *vs* anti-His or anti-FLAG mAb), resulting final cross-linked gp120 of 25 nM. Therefore, gp120 was used in an immune-complex rather than monomeric form, unless otherwise stated. In experiments to remove the carbohydrate moieties of gp120 by enzymatic digestion, we pre-treated the monomeric recombinant gp120 with endo-β-*N*-glucosaminidase H (EndoH; New England Biolabs, Cambridge, Mass, USA) by incubating recombinant gp120 supernatant with 25 KU of EndoH/ml for 16–18 hours, as described [39]. The extent of deglycosylation was then examined and confirmed by SDS-PAGE and Western blot analysis using polyclonal rabbit anti-gp120 antibodies (Fig. S2A). We then cross-linked the EndoH-treated monomeric gp120 (which had poly-His or FLAG at the COOH terminus) with anti-His or anti-FLAG Ab to form immune-complex (dimeric) gp120, as described above. The cross-linked EndoH-treated and -untreated gp120 were further examined by Western blot analysis in native non-reducing conditions, confirming the dimerization after addition of anti-His or anti-FLAG Ab (Fig. S2B). The binding of Endo-treated and -untreated dimeric gp120 to moDC was subsequently compared by flow cytometry, demonstrating that EndoH-treated gp120 had lost the binding to moDC (Fig. S2C).

### Pre-treatment of DC with receptor antagonists, mannan, and FcR blockade

To block gp120 binding to CD4 and chemokine receptors, DC were pre-treated at 4°C for 1 h with 10 µg/ml each anti-CD4 (RPA-T4) plus either anti-CCR5 (clone 2D7; mouse IgG2a) or anti-CXCR4 mAbs (clone 12G5, mouse IgG2a; all from BD PharMingen, San Diego, CA, USA). To block DC-SIGN, DC were similarly pre-treated with anti-DC-SIGN mAbs clone 120612 (mouse IgG2a; R & D Systems, MN, USA) plus DC28 (mouse IgG2a; AIDS Research and Reference Reagent Program, NIH) in combination at 10 µg/ml each. This combination of anti-DC-SIGN mAbs significantly inhibited both R5 and X4 gp120 binding to DC-SIGN-transfected 293 cells (Fig. S4A), and HIV-1 uptake into DC-SIGN-transfected THP-1 cells (a kind gift from Dr V. KewalRamani, HIV Drug Resistance Program, National Cancer Institute at Frederick, NIH, MD, USA; Fig. S4B). Soluble polyhistidin(HIS)-tagged ICAM-3-Fc chimeric protein (R & D Systems, Abingdon, UK; Fig. S4C), cross-linked with anti-His Ab (as above), was also used to pre-treat DC at 10 µg/ml for 1 h before use. After pre-treatment with receptor anatagonists, DC were washed and treated with or without recombinant gp120 as above or HIV(+) serum as below. In some experiments, we examined the effects of mannan in competing off gp120 binding and also the FcR blocking to enhance gp120 binding by pre-incubating moDC with mannan (50 µg/ml, Sigma, USA) or 30 µl FcR blocking reagent (Miltenyl Biotec, USA), or anti-DC-SIGN mAbs (as described above) for 30 minutes before pulse by EndoH-treated or –untreated gp120 pulsing with subsequent 3 days' coculture with CD40 Tf.

### Co-culture of gp120-DC with activated or naïve T cells

Autologous naïve CD4+ T cells were isolated from peripheral blood mononuclear cells (PBMCs) after two rounds of immunomagnetic depletion (Dynabeads, Dynal, Oslo, Norway) using a cocktail of mAbs against CD8, CD14, CD19, CD40, CD45RO, CD56 and HLA-DR (BD PharMingen, San Diego, CA, USA). The resultant cells were >98% CD4(+) CD45RA(+) (data not shown). Activated CD4+ T cells were prepared by treating naïve T cells with 10 ng/ml PMA and 1 µg/ml ionomycin (Sigma, St Louis, USA) for 24 h; they expressed high levels of CD40L (Fig. S3, left panel) whereas expression was very low or undetectable on naïve CD4 T cells (data not shown). 1×10^5^ recombinant gp120-primed moDC were co-cultured with 1×10^5^ activated or naïve CD4 T cells in 96-well plates for 3 d. Cells were then stained with anti-CD3-cychrome, Annexin V-FITC and Propidium Iodide (all from BD PharMingen, San Diego, USA). Apoptosis of DC was determined by the Annexin V-FITC and Propidium Iodide staining of the large granular cells in the FSC-SSC plot using a FACSCalibur instrument (Becton Dickinson, San Diego, CA, USA); these large granular cells were confirmed to lack expression of CD3 but to express high levels of DC-SIGN (Fig. S1A–B). For blocking CD40 ligand, activated CD4 T cells were treated with 10 µg/ml anti-CD40 ligand mAb (Alexis, Lausen, Switzerland) for 1 h at 4°C and washed before co-culture with gp120-DC.

### Culture of gp120-DC with CD40L transfectants, LPS, TNFα and IL-1β

1×10^5^ purified moDC were treated with cross-linked recombinant gp120 for 24 h, with or without prior receptor blockade, and co-cultured with 2.5×10^4^ CD40 ligand-transfected L (CD40L Tf) cells (a kind gift of Dr Yong-Jun Liu, MD Anderson Cancer Center, Texas, USA) in 96-well plates; the transfectants expressed high levels of CD40L, similar to those of activated CD4 T cells (Fig. S3, right panel). Before co-culture, the CD40L Tf cells were treated with 50 µg/ml mitomycin C (Sigma, St Louis, MO, USA) for 30 minutes at 37°C, in order to prevent cellular proliferation. After 3 days coculture, the cells were stained with cychrome-labeled anti-HLA-DR mAb (BD PharMingen, San Diego, USA), Annexin V-FITC and propidium iodide. Essentially all the moDC could be harvested by vigorous pipetting and re-suspension to separate them from the firmly adherent CD40L Tf cells; >98% of recovered cells expressed high levels of HLA-DR, whereas virtually no cells remaining in the wells expressed HLA-DR after being detached with EDTA (Fig. S2A). The survival of gp120-DC was determined by Annexin V and/or propidium iodide staining of cells in the HLA-DR(+) gated population, and by trypan blue staining (Fig. S2B). Co-culture of gp120-DC with mock transfected L cells was used as a control. Apoptosis of DC was also examined after culturing gp120-primed DC with 100 ng/ml each LPS (Salmonella typhosa, Sigma, St Louis, USA), TNF-α or IL-1β (Peprotech, NJ, USA) for 3 d.

### Isolation of DC-SIGN(+) cells from peripheral blood of HIV-uninfected [HIV(−)] and -infected [(HIV(+)] individuals

DC-SIGN(+) cells were isolated from HIV-1(−) individuals as described [9]. Briefly, 5×10^8^ PBMCs were collected from buffy coats of healthy individuals and the T, B and NK cells were removed by immunomagnetic depletion (Dynabeads, Dynal, Oslo, Norway) with a cocktail of anti-CD3/CD20/CD56 mAbs (BD PharMingen, San Diego, CA, USA). Cells were subsequently stained with PE-conjugated anti-CD14 (BD PharMingen, CA, USA) plus FITC-conjugated anti-DC-SIGN (AZN-D1, Beckman Coulter, CA, USA) mAbs, and subjected to positive sorting using a FACS Vantage (BD Bioscience, CA, USA); yields for DC-SIGN(+) cells ranged from 5–15×10^4^ cells (*i.e.*, 0.01–0.03% of the starting PBMC population). Cells were then cultured for 2 h in the presence of 5 mM EDTA to remove the bound mAb, washed, and cultured in fresh medium for additional 1–2 h. 2×10^4^ DC-SIGN(+) cells were then exposed to recombinant gp120_ADA_ and cocultured with CD40L transfectants before assessing cell viability as above. DC-SIGN(+) cells were similarly isolated from 3×10^8^ PBMCs of HIV-1(+) individuals after ethical review (Table S1); the yield ranged from 8–13×10^3^ cells (∼0.0027–0.0043% of starting PBMC). Cells were then treated and cocultured with CD40L Tf cells, or exposed to 100 ng/ml LPS, TNFα or IL-1β, prior to analysis [62] of cell viability as above.

### Treatment of moDC with serum and filtrates from HIV-1(+) individuals

The serum and PBMCs of HIV(−) individuals were obtained from blood collected for a genotyping study [62]. The whole HIV(+) serum was provided by the AIDS Research Laboratory, Department of Microbiology, Queen Mary Hospital, The University of Hong Kong, with approval of the Institutional Review Board. Before use, HIV-1(+) sera (Table S1) were centrifuged through ultra-centrifugal filters (Millipore, MA, USA) with a cut-off point of 100 kDa and fractions >100 kDa were reconstituted to the original volume with fresh medium as previously described [27]. Immunoprecipitation (IP) with anti-gp120 mAbs was performed as described [27] and resulted in over 80% reduction of the p24 level (Fig. S7). Where indicated, the >100 kDa fraction was further fractionated by centrifugation through ultra-centrifugal filters with a cut-off point of 1000 kDa after which the <1000 kDa and >1000 kDa portions were each reconstituted to the original volume with fresh medium. 1×10^5^ moDC generated from PBMC of HIV(−) individuals were exposed to the respective HIV(+) serum fractions (above) for 24 h at room temperature, washed, and then co-cultured with CD40L Tf or autologous activated CD4 T cells, or 100 ng/ml LPS, for 3 d. After recovery from the adherent CD40L Tf cells (Fig. S2A), apoptosis of moDC was determined by Annexin V and PI staining as above. Alternatively, for biosafety reasons after co-culture with activated CD4 T cells, apoptosis was determined by terminal deoxynucleotidyl transferase (TdT) dUTP nick end labelling (TUNEL) assay using the In-Situ Cell Death Detection Kit (Roche Diagnostics, Inc.), according to manufacturer's instructions; the recovered moDC were fixed, permeabilized and subjected to TUNEL reaction mix incubation at 37°C for 1 h, followed by cytometric analysis.

### Western blotting and siRNA transfection

Purified moDC were pulsed with or without whole HIV-1(+) sera or recombinant gp120_ADA_ for 24 h, washed, and respectively co-cultured with CD40L Tf cells or LPS, TNFα or IL-1β for 3 d, as described before. MoDC could be separated from CD40L Tf and recovered by pipetting and re-suspension in the co-culture wells. In preliminary experiments, the recovered cells were confirmed to express high MHC class II (HLA-DR), while the CD40L Tf (which could be subsequently detached by treatment with 5 mM EDTA for 15 min) expressed no or little MHC class II (Fig. S4B). The recovered moDCs were then washed with ice-cold PBS, and lysed with buffer containing a proteinase inhibitor cocktail (Sigma, St Louis, USA). 30 µg of total cell lysate protein aliquots were loaded onto 12% polyacrylamide gels and subjected to SDS-PAGE analysis. Gels were blotted onto Immobilon P membranes (Millipore, Bedford, Mass., USA), blocked in Tris-buffered saline (TBS) containing 0.05% Tween 20 (TBS-T) and 5% milk, and probed with primary mAbs against human Bcl-2, Bcl-xL (Zymed laboratories Inc., CA, USA, both 1∶200 dilution), phospho-Akt (Ser-473), phospho-c-Raf (Ser338), or rabbit polyclonal antibodies to phospho-ASK1 (Thr845) (1∶1000, Cell Signaling, USA), or β-actin (1∶1000, Santa Cruz, Ca, USA) overnight at 4°C. Membranes were then washed and incubated with horseradish peroxidase-conjugated secondary antibody (1∶5000; Santa Cruz, CA, USA) for 1 h at room temperature. After washing, membranes were developed with an ECL enhanced chemiluminescence Western blot kit and exposed to Hyperfilm (both from Amersham Pharmacia Biotech, Inc., Piscataway, N.J., USA) at room temperature. The rabbit polyclonal anti-p-ASK1(Thr845) Ab was confirmed to detect p-ASK1 expression of HEK293 cells transfected with human ASK-1 (Fig. S12). Transfection of moDC with siRNA for human ASK1 or control siRNA (20 µM each, Santa Cruz, USA) was performed as described [63] by Oligofectamine, followed by protocols according to manufacturer's instruction (Invitrogen, Carlsbad, CA, USA). Day 5–6 moDC were transfected 1 day before pulsing with immune-complex gp120.

### Statistical analysis

Data were presented as mean ± standard deviations and analyzed by ANOVA using SPSS 10 software.

## Supporting Information

Figure S1 **moDC in coculture with autologous activated CD4 T cells can be identified as a large granular CD3-negative, DC-SIGN-positive population by flow cytometry.** (***A***) After vigorous pipetting and re-suspension of the cells in the DC-T coculture wells, consistently <5% of the cells in the larger and more granular population (gated in R1, left panel) expressed CD3 (right upper panel). In contrast, consistently >95% of the smaller and less granular cells (gated in R2) expressed CD3. Data are representative of 3 experiments. Solid line, anti-CD3; dashed line, isotype control. (***B***) more than 90% of R1 population (gated, left panel) expressed DC-SIGN (right panel), while only 4.3% of R2 cells did so. Therefore, cells in R1 are mostly moDCs, whereas those in R2 are mostly T cells. Data are representative of 6 experiments. (***C***) MoDC which were pulsed with cross-linked (immune-complex) gp120 and cocultured with autologous activated CD4 T cells for 2 to 4 days were analyzed for their viability by AV and PI staining. Results showed that the percentage of AV(+)PI(−) cells (right lower quadrant) increased from d2 to d3 but decreased from d3 to d4, whereas AV(+)PI(+) cells (right upper quadrant) increased from d2 to d3, and further increased to d4. The total AV+ cells (including both AV+PI− and AV+PI+ cells), nevertheless, remained relatively consistent between d3 and d4 (58.7% *vs* 58.4%). This finding is in line with results of Fig. 1C (of the text) and indicates that as incubation time increases, some cells at early apoptotic (AV+PI−) phase would become late apoptotic (AV+PI+) cells. Results are representative of 3 experiments.
(TIF)

Figure S2 **EndoH treatment of recombinant gp120 abolished its binding to moDC.** (***A***) Recombinant gp120_ADA_ supernatant was treated overnight with 25 KU of EndoH/ml as described (Hong PWP et al, *J Virol* 2002;76:12855–12865), and the treated and the untreated gp120_ADA_ supernatant were subjected to Western blot assay by polyclonal rabbit anti-gp120 Ab (Sino Biological Inc). The untreated gp120 had a MW≈120 kDa, whereas the EndoH-treated one had MW≈80 kDa. (***B***) EndoH-untreated recombinant gp120_ADA_ and gp120_HXBc2_ were cross-linked with anti-His or anti-FLAG Ab, as described in materials and methods, and subsequently subjected to native non-reducing Western blot analysis (as described by Hong PWP et al, *J Virol* 2002;76:12855–12865). The cross-linked gp120 had MW≈410–420 kDa (lane 1 & 2, ***left panel***), and the MW of monomeric gp120 was ≈120 kDa (lane 3 & 4). The cross-linked deglycosylated (EndoH-treated) gp120 were in lanes 5 & 6 (≈330–340 kDa), and monomeric EndoH-treated gp120 were in lanes 7 & 8 (≈80 kDa). In a separate gel, the MW of anti-His or anti-FLAG Abs used for cross linking (as detected by goat polyclonal anti-mouse IgG Ab; Genscript, USA) was ≈170–180 kDa (lane 9). Flow cytometry of EndoH-treated or -untreated cross-linked gp120_ADA_ binding on moDC was shown in the ***right panel***. Briefly, 5×10^5^ moDC were fixed with formalin first and incubated by EndoH-treated or -untreated gp120_ADA_ cross-linked by mouse anti-His Ab for 30 min. After wash cells were incubated with the FITC-conjugated goat polyclonal anti-mouse IgG antibody (BioLegend) for 30 min. Isotype control antibody (black line) was goat control Ig (Abcam). Data are representative of 3 independent experiments.
(TIF)

Figure S3 **Anti-DC-SIGN mAbs inhibited binding of HIV-1 gp120 and uptake of HIV-1 virions by DC-SIGN transfectants which bound soluble ICAM-3-Fc chimeric recombinant protein.** (***A***) DC-SIGN-transfected or mock-transfected 293 cells were pre-treated without (as a control) or with anti-DC-SIGN mAb clones 120612 and DC28, individually or in combination (10 µg/ml each), for 1 h at 4°C. After wash, cells were incubated for 1 h at 4°C with 10 µg/ml recombinant gp120_ADA_ cross-linked by FITC-conjugated anti-His mAb, or with recombinant gp120_IIIB_ and gp120_MN_ cross-linked by 2G12 mAb (NIH AIDS Research and Reference Reagent Program) as indicated, washed and analysed by flow cytometry. Shaded area, binding of recombinant gp120; solid line, gp120 binding after pre-treatment with anti-DC-SIGN mAb(s); dashed line, binding of gp120 to mock transfectants. Note that the most effective inhibition, though still incomplete, of gp120 binding was seen with the combination of anti-DC-SIGN mAbs, while DC28 was more effective than 120612 when tested individually. Data are representative of 3 experiments. (***B***) 5×10^5^ DC-SIGN-transfected (Tf) or mock-transfected THP-1 cells were incubated with HIV-1_89.6_ (p24 = 1.5 ng; from NIH AIDS Research and Reference Reagent Program) in a total volume of 400 µl for 3 hours at 37°C to allow cellular adsorption of the virus and viral replication. The cells were pre-treated without or with a combination of anti-DC-SIGN mAbs (clone 120612 plus DC28) at room temperature for 30 minutes prior to exposure to virus supernatant and during subsequent culture. Cells were then washed extensively to remove unbound virus, lysed in 0.5% Triton X-100, and the lysates were subjected to analysis using p24 ELISA kits (Coulter, FL, USA). Data are expressed as mean±SD of 3 experiments.
(TIF)

Figure S4 **CD40L-transfected L cells expressed CD40L with levels similar to activated CD4 T cells, and gp120-DCs can be recovered from adherent CD40 ligand-transfected (CD40L Tf) cells.** (***A***) Activated CD4 T cells (treated with 10 µg/ml PMA and 1 µg/ml ionomycin for 24 h) or CD40L Tf cells were incubated with 10 µg/ml anti-CD40L mAb (Alexis, Lausen, Switzerland) or isotype control and analyzed by flow cytometry using a FACSCalibur (Becton Dickinson, San Diego, CA, USA). Solid line, anti-CD40L mAb; dashed line, isotype control. Data are representative of 4 experiments. (***B***) Cells recovered from DC+CD40L Tf coculture expressed high levels of HLA-DR. Briefly, moDCs were treated with cross-linked recombinant gp120_ADA_ and subsequently co-cultured with CD40L Tf cells for 3 days. The non- and weakly-adherent cells were harvested by pipetting and resuspension and subsequently labeled by anti-HLA-DR mAb (R & D systems) and subjected to flow cytometric analysis. Virtually all the recovered (non-adherent) cells expressed HLA-DR (left panel). The remaining adherent cells that had been detached with EDTA expressed little HLA-DR (right panel), confirming that virtually all DCs had been recovered for analysis. Data are representative of 3 experiments, and results were similar for the cocultures of CD40L Tf and DCs pulsed with gp120_ADA_ or HIV-1(+) serum (not shown).
(TIF)

Figure S5 **Trypan blue staining of moDC that were treated with anti-His cross-linked gp120_ADA_ or anti-His mAb alone (control DC) and recovered from co-culture with CD40L Tf after 3 days.** Data represent mean ± SD from 3 experiments; ***p<0.001.
(TIF)

Figure S6 **Soluble recombinant ICAM-3-Fc chimeric protein (sICAM-3) bound to DC-SIGN-transfectants and pre-treatment with receptor antagonist mAbs alone did not sensitize DC for CD40L-mediated apoptosis.** (***a***) DC-SIGN-transfected or mock-transfected 293 cells were incubated with 10 µg/ml soluble ICAM-3-Fc chimeric protein (cross-linked with anti-His mAb) or with anti-His mAb alone, for 1 h at 4°C. After wash, cells were incubated with FITC-conjugated rabbit-anti-mouse antibody (DAKO, Denmark) for 1 h at 4°C and analysed by flow cytometry. Solid line, cross-linked sICAM-3-Fc; dashed line, anti-His mAb. Data are representative of 3 experiments. (***b***) moDC were treated for 24 h with combinations of anti-His plus anti-FLAG mAbs (panel ***A***) which were used to cross-link the gp120, anti-CD4 (clone RPA-T4) plus anti-CCR5 (clone 2D7) mAbs (panel ***B***), anti-CD4 plus anti-CXCR4 (clone 12G5) mAbs (panel ***C***), anti-DC-SIGN (clones 120612 plus DC28) mAbs (panel ***D***), or with cross-linked gp120_ADA_ or gp120_HXBc2_ respectively (cross-linked with anti-His or anti-FLAG mAbs; panel ***E*** and ***F*** respectively), and co-cultured with CD40L transfectants for 3 days. All antibodies and gp120 were used at 25 nM and 50 nM each. Flow cytometric analysis confirmed little apoptosis of the mAb-treated moDC (panels ***A***–***D***) compared to the gp120-DC (panels ***E*** & ***F***). Data are representative of 3 experiments. In further experiments, treatment with a combination of the three anti-CD4/CCR5/CXCR4 mAbs did not induce CD40L Tf-mediated DC apoptosis either (data not shown).
(TIF)

Figure S7 **moDCs treated with HIV(+) sera can be identified as a distinctly smaller-sized CD3-negative population compared to control moDC.** MoDC were treated with normal AB serum (Control DC) or with HIV-1(+) serum [HIV(+) serum-DC] with viral loads >400,000/ml (Table S1) for 24 h, and co-cultured with autologous activated CD4 T cells for 3 d. Cells were then harvested and subjected to flow cytometric analysis. In contrast to control moDC (R1 in left panel; compare to Fig. S1A), the sera-treated moDCs were identified as a distinctly smaller CD3-negative population (round circle in the middle panel), indicative of the induction of apoptosis. Data are representative of 3 experiments.
(TIF)

Figure S8 **Immunoprecipitation (IP) of gp120 reduced p24 levels in supernatants of** ***in vitro*** **propagated live virus.** IP was performed by adding 10 µg/ml each of anti-gp120 mAbs 2G12 and IgG1b12 (NIH AIDS Research and Reference Reagent Program) into 2 ml supernatants of *in vitro* propagated HIV-1_BaL_ (BaL) or HIV-1_IIIB_ (IIIB) virus. Solutions were mixed on ice for 1 hour and 20 µg of protein A sepharose (Sigma-Aldrich, St Louis, USA) in PBS was added for additional 1 hour. Protein A beads were then removed by centrifugation. Two further rounds of protein A sepharose depletion were similarly performed before retrieval of the depleted serum for determination of p24 levels by ELISA kit (Coulter, FL, USA). Data are expressed as mean ± SD of 3 experiments.
(TIF)

Figure S9 **Pre-treatment with mannan abolished CD40L-mediated apoptosis of moDC pulsed by HIV serum and FcR blocking of moDC enhanced the CD40L-mediated death of HIV serum-pulsed DC.** (***A*** & ***B***) 50KU of EndoH (in 100 µl) and 100 µl of protease inhibitors (cOmplete ULTRA tablet, EDTA-free; from Roche; in order to protect the integrity of EndoH after addition into the serum) were mixed into 1 ml of HIV(+) serum (with a viral titer >400,000/ml; Table S1) for 5–10 minutes before being ultra-centrifuged to obtain the 100–1000 kDa fraction (as described in “materials and methods”). DC were then pulsed by such EndoH-treated or untreated HIV serum (100–1000 kDa fraction) after being pre-treated with or without mannan or FcR blocking reagent (as described in Fig. 2E of the text), or anti-DC-SIGN mAbs, and subsequently cocultured with CD40L Tf. After 3 days, DC were detached and recovered, and underwent AV/PI staining. DC pulsed by normal AB serum were used a control. Results revealed that DC pulsed by EndoH-treated HIV(+) serum had significant reduction in CD40L-mediated apoptosis (down to control levels), when compared with cells pulsed by EndoH-untreated HIV serum. Furthermore, consistently as in the use of recombinant gp120, FcR blocking itself did not induce excessive apoptosis of control DC (which were pulsed with normal AB serum), and FcR blockade of DC prior to pulse with HIV serum further increased the extent of CD40L-mediated apoptosis (P<0.05, in comparison to no FcR blockade). In addition, compared with DC-SIGN blockade which partially prevented DC death (p<0.05 compared to DC without DC-SIGN blockade), pre-treatment of DC with mannan further reduced the CD40L-mediated DC apoptotic (*p<0.05 compared to DC-SIGN blockade). Representative of 3 independent experiments was shown in ***A***, and the % of total AV(+) cells in each condition was expressed as mean ± SD in ***B***.
(TIF)

Figure S10 **Freshly-isolated DC-SIGN(+) cells from blood expressed CD40 and CD11c.** DC-SIGN(+) cells (gated in the left panel) were isolated from the CD14(+) subset of PBMCs as described in the Materials and Methods. Cells were incubated with FITC-conjugated anti-CD40 or anti-CD11c mAbs (BD PharMingen, CA, USA) and analyzed by flow cytometry (middle and right panels). In line with a previous report (Engering A et al, Blood 2002;100:1780–1786), essentially all DC-SIGN(+) cells expressed CD11c and CD40. Data are representative of 3 experiments.
(TIF)

Figure S11 **Cross-linked gp120 modulates the expression of surface molecules typically associated with DC maturation in a DC-SIGN-dependent manner but is inefficient compared with maturation by conventional factors.** (***A***) 1×10^6^ moDC were cultured for 24 h at 37°C in the presence of 10 µg/ml monomeric gp120_ADA_ (left column), anti-His cross-linked gp120_ADA_ (10 µg/ml, middle column), or anti-His cross-linked gp120_ADA_ with DC-SIGN blockade (10 µg/ml each of anti-DC-SIGN mAbs clone 12612 and DC28; right column). Results indicated that immune-complex gp120 indeed could induce phenotypic maturation of moDC, as manifested by clear upregulation of CD80, CD83, CD86, and CCR7, and downregulation of CCR5. Such modulation could be in part prevented by pre-treatment by anti-DC-SIGN mAbs (right column). The change in CD40 and HLA-DR expression was not as remarkable as others. Data are representative of 5 experiments. (***B***) moDC were treated with dimeric (cross-linked) gp120 (10 µg/ml; immune-complex/gp120 or IC/gp120) or conventional DC maturation factors as similarly described by Shan M *et al*, PLoS Pathogens 2007;3:1637–1650, *ie*,, exposure to a mixture of 10 ng/ml LPS, 25 ng/ml TNFα, 10 ng/ml IL-1β, and simultaneous coculture with CD40L (as described in “materials and methods”) for 24 h. DCs with no treatment were used as a control (control DCs). After treatment, DC were subjected to flow cytometric analysis of surface expression of costimulatory and maturation markers. Compared with conventional maturation, IC/gp120 induced inefficient modulation of moDCs in the upregulation of CD80, CD86, CD83, CCR7, as well as in the downregulation of CCR5. In contrast, the upregulation of MHC class II appeared to be less affected. Data are representative of 3 independent experiments.
(TIF)

Figure S12 **Expression of the anti-apoptotic Bcl-2 and Bcl-xL was reduced after co-culture of gp120-primed moDCs with CD40L transfectants, and rabbit polyclonal anti-ASK1 Ab detected p-ASK1 expression in ASK1-transfected cells.** (***A***) moDC were treated with cross-linked gp120_ADA_ (gp120-DC) or anti-His mAb (control DC) followed by co-culture with CD40L Tf. After 3 d, DC were harvested from and lysed, and cellular protein was subjected to western blotting for the indicated proteins. Data are representative of 3 experiments. (***B***) moDC were treated with HIV-1(+) serum (RNA copy number>400,000/ml) with or without pre-treatment by anti-DC-SIGN mAbs, as described in “materials and methods”, prior to coculture with CD40L transfectants as described in panel ***A***, and cellular protein was extracted and subjected to western blotting for the indicated proteins. Data are representative of 3 experiments. (***C***) Human ASK1 (Ichijo H et al, *Science* 1997;275:90–94) was generated in pcDNA3 vector, as described (Won M et al, *Cell Death and Differentiation* 2010;17:1830–1841), and transiently transfected into HEK293 cells by lipofecatmine PLUS, according to manufacturer's instructions. After ≈36 hours, cell were lysed and subjected to Western blot assay with rabbit polyclonal anti-ASK1 Ab (Phospho-ASK1 (Thr845) antibody, #3765, Cell Signaling, USA). Results confirmed ASK1 expression with a molecular weight ≈160 kDa, which served as a positive control for p-ASK1 expression in Fig. 7A. Data are representative of 3 independent experiments.
(TIF)

Table S1 **Viral RNA copy numbers in the sera of HIV-1 infected individuals used for this study.** Patient viral RNA copy numbers were retrieved from archived information. The following individual or pooled patient (#) samples were used to obtain the data shown in the respective figures: Figures 3A #9; 3B #7–10; 3C #10; 3D #5–7 and #10; 4A #1–4 and 5–7; 4B #6, 4C #6–10; 5C #3, 5C–D #1–4; 6D #5–8; 6E #9; and 6F #6 plus #8–10.
(TIF)
