# Supplementary figures and images for: Binding of HIV-1 gp120 to DC-SIGN Promotes ASK-1-Dependent Activation-Induced Apoptosis of Human Dendritic Cells
Source: PLoS Pathog. 2013 Jan 31;9(1):e1003100. doi: 10.1371/journal.ppat.1003100 (PMC3561151; doi:10.1371/journal.ppat.1003100)

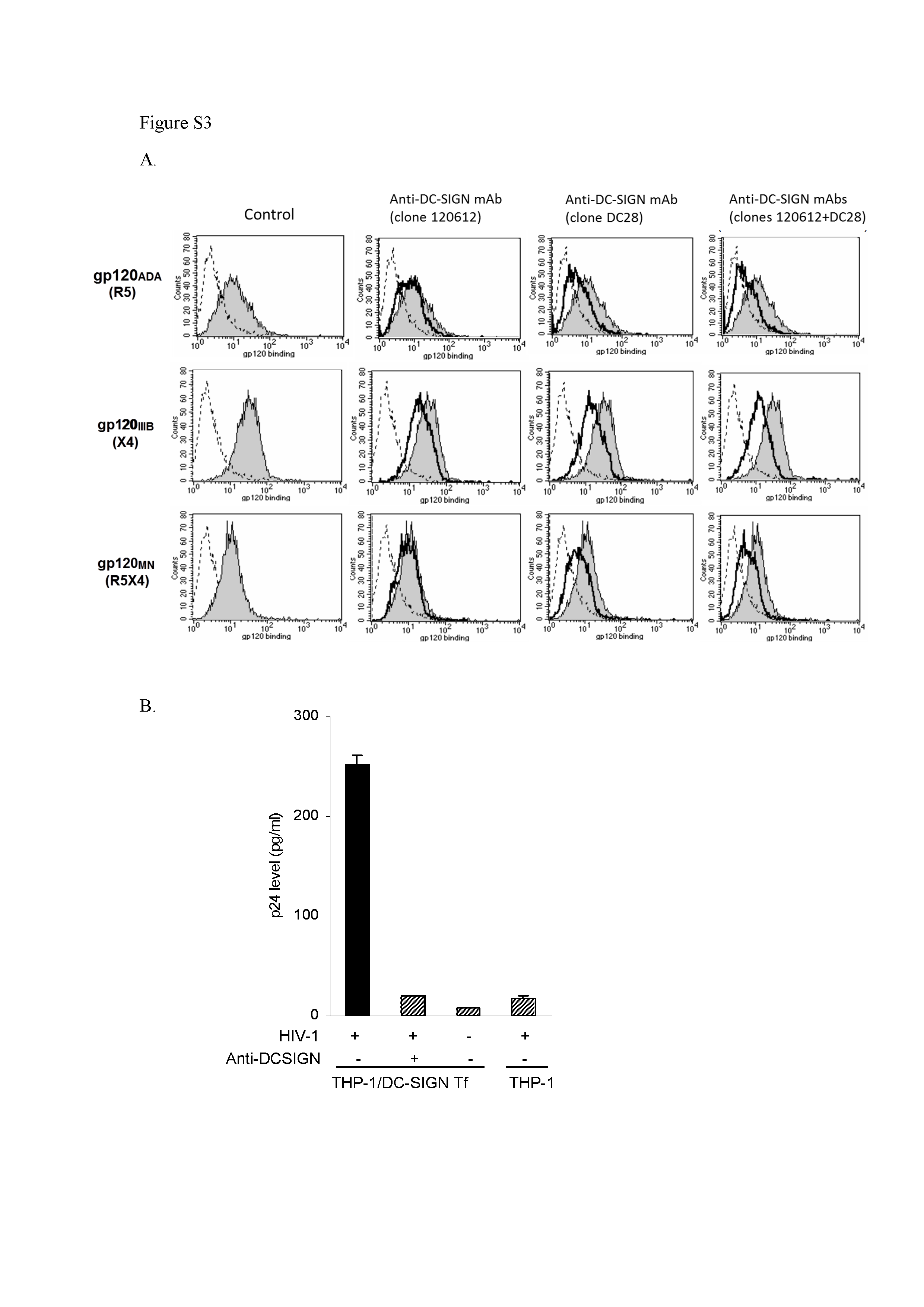

Supplement: Figure S3 — Anti-DC-SIGN mAbs inhibited binding of HIV-1 gp120 and uptake of HIV-1 virions by DC-SIGN transfectants which bound soluble ICAM-3-Fc chimeric recombinant protein. (A) DC-SIGN-transfected or mock-transfected 293 cells were pre-treated without (as a control) or with anti-DC-SIGN mAb clones 120612 and DC28, individually or in combination (10 µg/ml each), for 1 h at 4°C. After wash, cells were incubated for 1 h at 4°C with 10 µg/ml recombinant gp120ADA cross-linked by FITC-conjugated anti-His mAb, or with recombinant gp120IIIB and gp120MN cross-linked by 2G12 mAb (NIH AIDS Research and Reference Reagent Program) as indicated, washed and analysed by flow cytometry. Shaded area, binding of recombinant gp120; solid line, gp120 binding after pre-treatment with anti-DC-SIGN mAb(s); dashed line, binding of gp120 to mock transfectants. Note that the most effective inhibition, though still incomplete, of gp120 binding was seen with the combination of anti-DC-SIGN mAbs, while DC28 was more effective than 120612 when tested individually. Data are representative of 3 experiments. (B) 5×10^5^ DC-SIGN-transfected (Tf) or mock-transfected THP-1 cells were incubated with HIV-189.6 (p24 = 1.5 ng; from NIH AIDS Research and Reference Reagent Program) in a total volume of 400 µl for 3 hours at 37°C to allow cellular adsorption of the virus and viral replication. The cells were pre-treated without or with a combination of anti-DC-SIGN mAbs (clone 120612 plus DC28) at room temperature for 30 minutes prior to exposure to virus supernatant and during subsequent culture. Cells were then washed extensively to remove unbound virus, lysed in 0.5% Triton X-100, and the lysates were subjected to analysis using p24 ELISA kits (Coulter, FL, USA). Data are expressed as mean±SD of 3 experiments. (TIF) [file ppat.1003100.s003.tif]

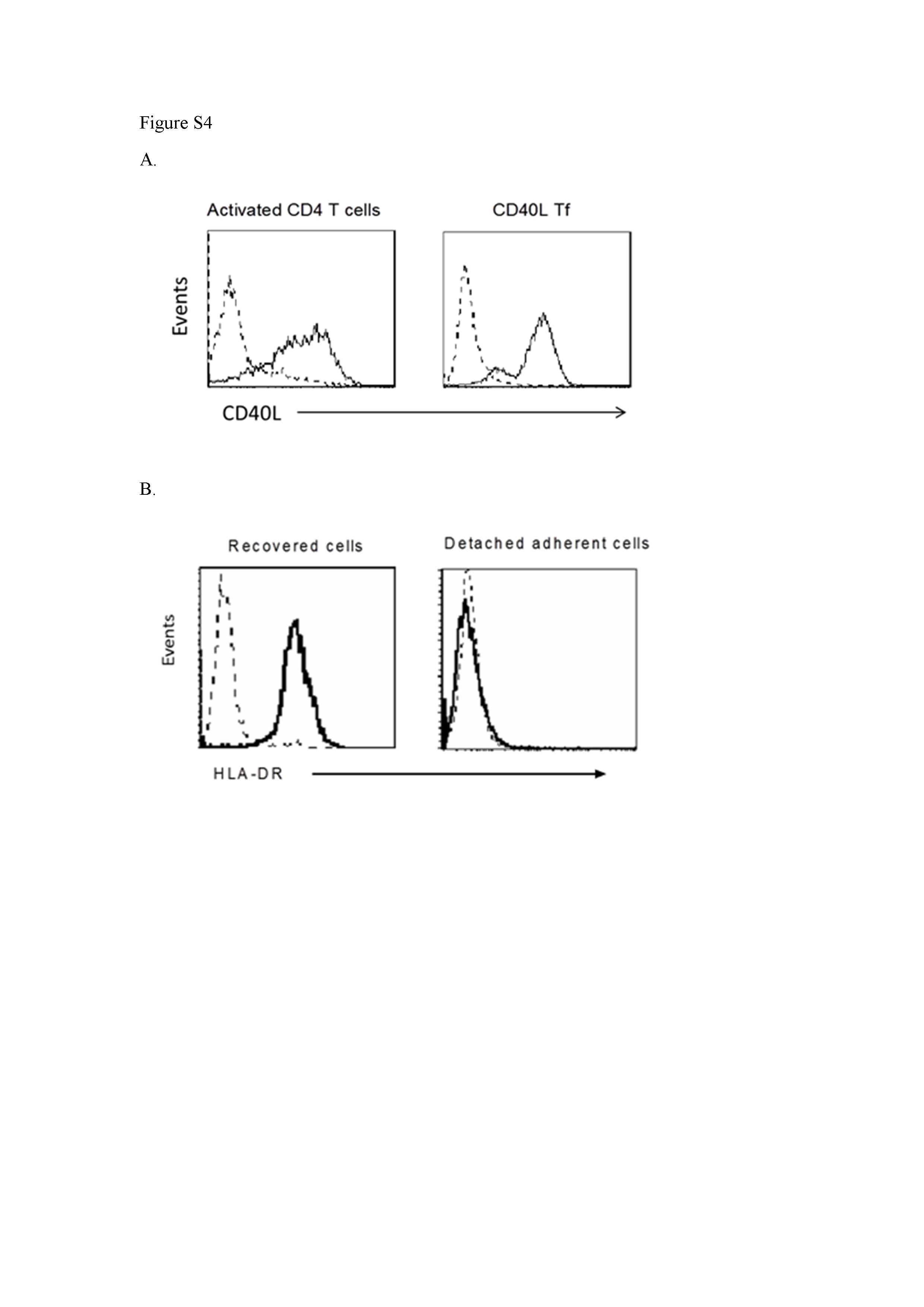

Supplement: Figure S4 — CD40L-transfected L cells expressed CD40L with levels similar to activated CD4 T cells, and gp120-DCs can be recovered from adherent CD40 ligand-transfected (CD40L Tf) cells. (A) Activated CD4 T cells (treated with 10 µg/ml PMA and 1 µg/ml ionomycin for 24 h) or CD40L Tf cells were incubated with 10 µg/ml anti-CD40L mAb (Alexis, Lausen, Switzerland) or isotype control and analyzed by flow cytometry using a FACSCalibur (Becton Dickinson, San Diego, CA, USA). Solid line, anti-CD40L mAb; dashed line, isotype control. Data are representative of 4 experiments. (B) Cells recovered from DC+CD40L Tf coculture expressed high levels of HLA-DR. Briefly, moDCs were treated with cross-linked recombinant gp120ADA and subsequently co-cultured with CD40L Tf cells for 3 days. The non- and weakly-adherent cells were harvested by pipetting and resuspension and subsequently labeled by anti-HLA-DR mAb (R & D systems) and subjected to flow cytometric analysis. Virtually all the recovered (non-adherent) cells expressed HLA-DR (left panel). The remaining adherent cells that had been detached with EDTA expressed little HLA-DR (right panel), confirming that virtually all DCs had been recovered for analysis. Data are representative of 3 experiments, and results were similar for the cocultures of CD40L Tf and DCs pulsed with gp120ADA or HIV-1(+) serum (not shown). (TIF) [file ppat.1003100.s004.tif]

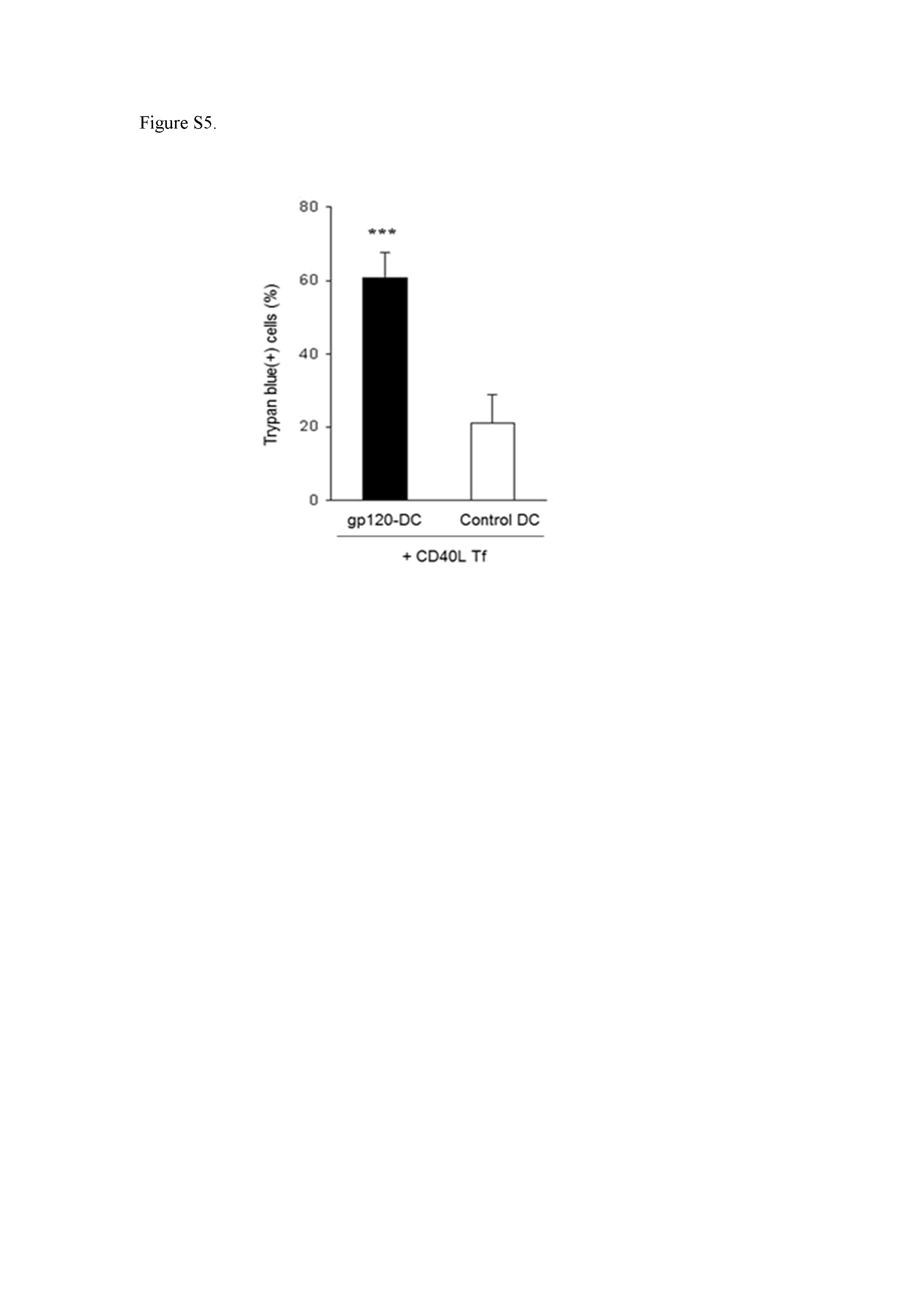

Supplement: Figure S5 — Trypan blue staining of moDC that were treated with anti-His cross-linked gp120ADA or anti-His mAb alone (control DC) and recovered from co-culture with CD40L Tf after 3 days. Data represent mean ± SD from 3 experiments; ***p<0.001. (TIF) [file ppat.1003100.s005.tif]

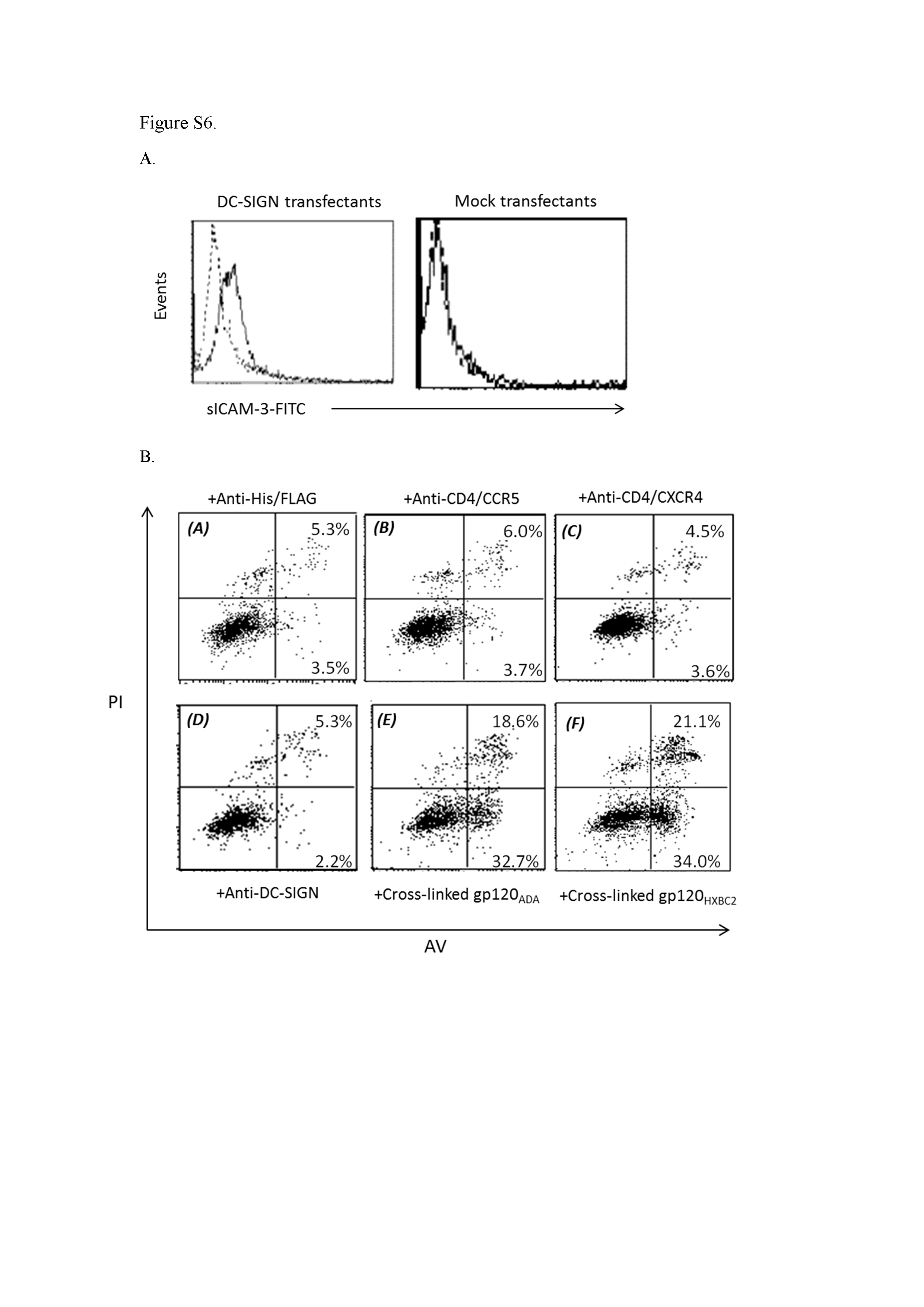

Supplement: Figure S6 — Soluble recombinant ICAM-3-Fc chimeric protein (sICAM-3) bound to DC-SIGN-transfectants and pre-treatment with receptor antagonist mAbs alone did not sensitize DC for CD40L-mediated apoptosis. (a) DC-SIGN-transfected or mock-transfected 293 cells were incubated with 10 µg/ml soluble ICAM-3-Fc chimeric protein (cross-linked with anti-His mAb) or with anti-His mAb alone, for 1 h at 4°C. After wash, cells were incubated with FITC-conjugated rabbit-anti-mouse antibody (DAKO, Denmark) for 1 h at 4°C and analysed by flow cytometry. Solid line, cross-linked sICAM-3-Fc; dashed line, anti-His mAb. Data are representative of 3 experiments. (b) moDC were treated for 24 h with combinations of anti-His plus anti-FLAG mAbs (panel A) which were used to cross-link the gp120, anti-CD4 (clone RPA-T4) plus anti-CCR5 (clone 2D7) mAbs (panel B), anti-CD4 plus anti-CXCR4 (clone 12G5) mAbs (panel C), anti-DC-SIGN (clones 120612 plus DC28) mAbs (panel D), or with cross-linked gp120ADA or gp120HXBc2 respectively (cross-linked with anti-His or anti-FLAG mAbs; panel E and F respectively), and co-cultured with CD40L transfectants for 3 days. All antibodies and gp120 were used at 25 nM and 50 nM each. Flow cytometric analysis confirmed little apoptosis of the mAb-treated moDC (panels A–D) compared to the gp120-DC (panels E & F). Data are representative of 3 experiments. In further experiments, treatment with a combination of the three anti-CD4/CCR5/CXCR4 mAbs did not induce CD40L Tf-mediated DC apoptosis either (data not shown). (TIF) [file ppat.1003100.s006.tif]

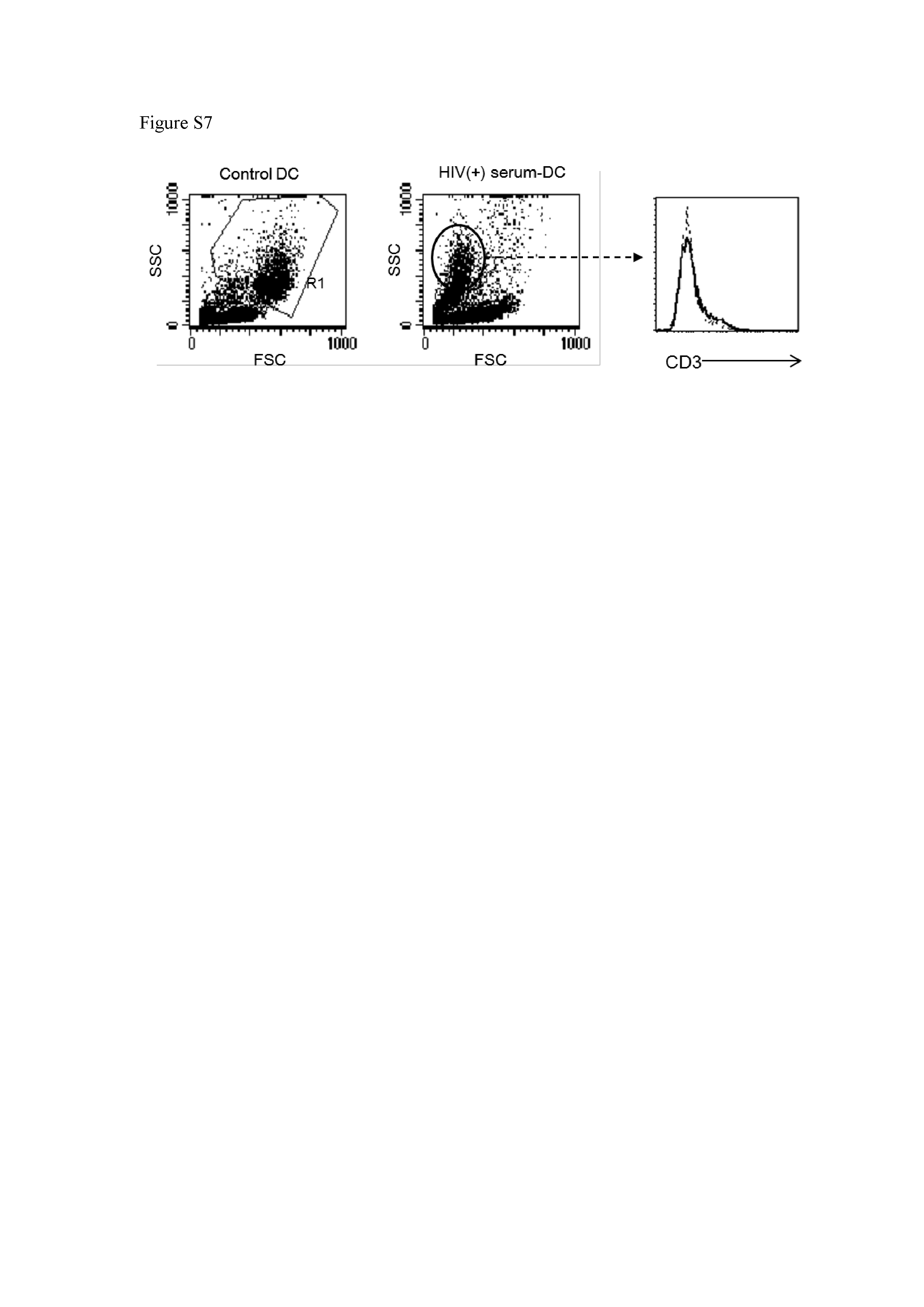

Supplement: Figure S7 — moDCs treated with HIV(+) sera can be identified as a distinctly smaller-sized CD3-negative population compared to control moDC. MoDC were treated with normal AB serum (Control DC) or with HIV-1(+) serum [HIV(+) serum-DC] with viral loads >400,000/ml (Table S1) for 24 h, and co-cultured with autologous activated CD4 T cells for 3 d. Cells were then harvested and subjected to flow cytometric analysis. In contrast to control moDC (R1 in left panel; compare to Fig. S1A), the sera-treated moDCs were identified as a distinctly smaller CD3-negative population (round circle in the middle panel), indicative of the induction of apoptosis. Data are representative of 3 experiments. (TIF) [file ppat.1003100.s007.tif]

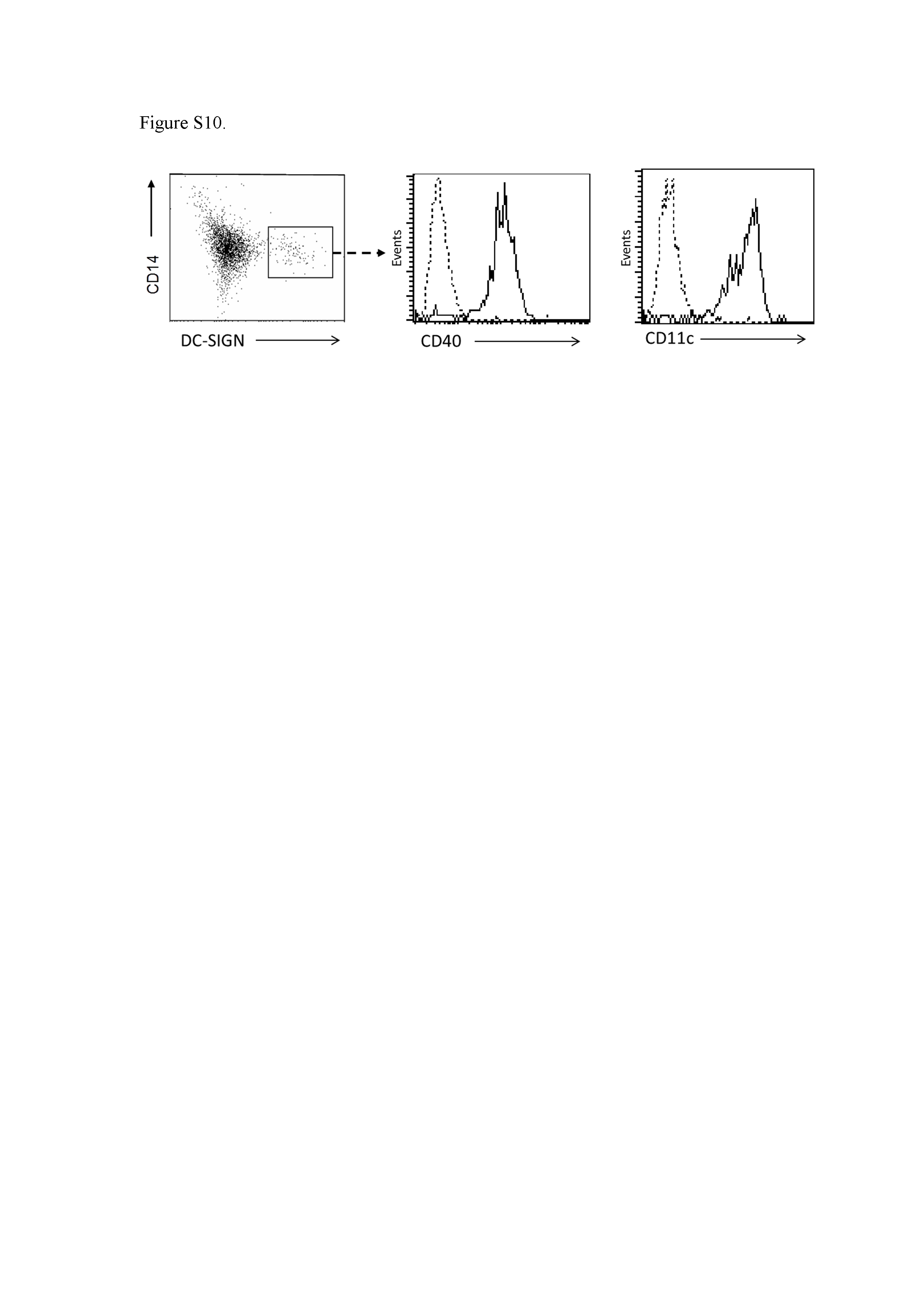

Supplement: Figure S10 — Freshly-isolated DC-SIGN(+) cells from blood expressed CD40 and CD11c. DC-SIGN(+) cells (gated in the left panel) were isolated from the CD14(+) subset of PBMCs as described in the Materials and Methods. Cells were incubated with FITC-conjugated anti-CD40 or anti-CD11c mAbs (BD PharMingen, CA, USA) and analyzed by flow cytometry (middle and right panels). In line with a previous report (Engering A et al, Blood 2002;100:1780–1786), essentially all DC-SIGN(+) cells expressed CD11c and CD40. Data are representative of 3 experiments. (TIF) [file ppat.1003100.s010.tif]

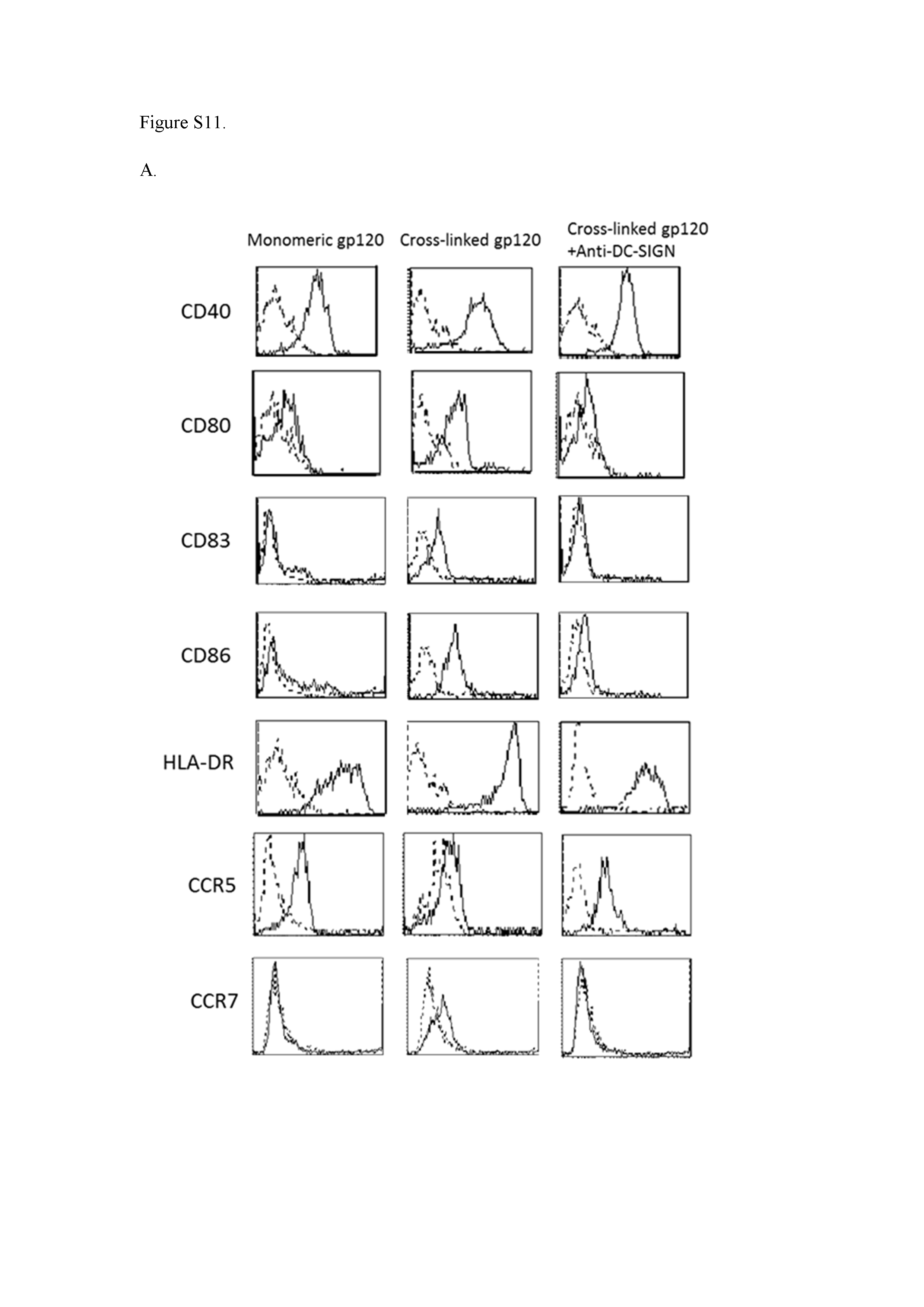

Supplement: Figure S11 — Cross-linked gp120 modulates the expression of surface molecules typically associated with DC maturation in a DC-SIGN-dependent manner but is inefficient compared with maturation by conventional factors. (A) 1×10^6^ moDC were cultured for 24 h at 37°C in the presence of 10 µg/ml monomeric gp120ADA (left column), anti-His cross-linked gp120ADA (10 µg/ml, middle column), or anti-His cross-linked gp120ADA with DC-SIGN blockade (10 µg/ml each of anti-DC-SIGN mAbs clone 12612 and DC28; right column). Results indicated that immune-complex gp120 indeed could induce phenotypic maturation of moDC, as manifested by clear upregulation of CD80, CD83, CD86, and CCR7, and downregulation of CCR5. Such modulation could be in part prevented by pre-treatment by anti-DC-SIGN mAbs (right column). The change in CD40 and HLA-DR expression was not as remarkable as others. Data are representative of 5 experiments. (B) moDC were treated with dimeric (cross-linked) gp120 (10 µg/ml; immune-complex/gp120 or IC/gp120) or conventional DC maturation factors as similarly described by Shan M et al, PLoS Pathogens 2007;3:1637–1650, ie,, exposure to a mixture of 10 ng/ml LPS, 25 ng/ml TNFα, 10 ng/ml IL-1β, and simultaneous coculture with CD40L (as described in “materials and methods”) for 24 h. DCs with no treatment were used as a control (control DCs). After treatment, DC were subjected to flow cytometric analysis of surface expression of costimulatory and maturation markers. Compared with conventional maturation, IC/gp120 induced inefficient modulation of moDCs in the upregulation of CD80, CD86, CD83, CCR7, as well as in the downregulation of CCR5. In contrast, the upregulation of MHC class II appeared to be less affected. Data are representative of 3 independent experiments. (TIF) [file ppat.1003100.s011.tif]

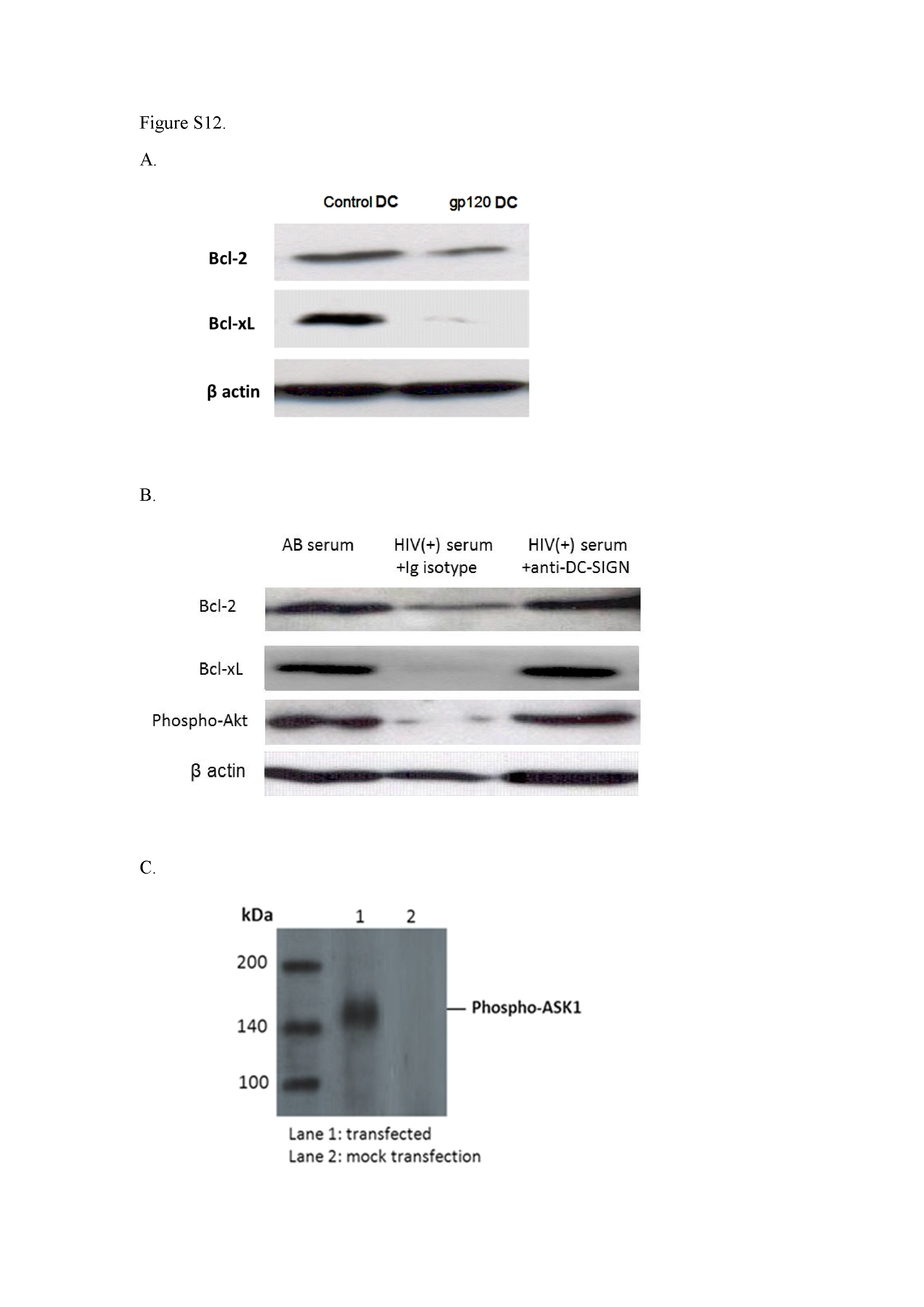

Supplement: Figure S12 — Expression of the anti-apoptotic Bcl-2 and Bcl-xL was reduced after co-culture of gp120-primed moDCs with CD40L transfectants, and rabbit polyclonal anti-ASK1 Ab detected p-ASK1 expression in ASK1-transfected cells. (A) moDC were treated with cross-linked gp120ADA (gp120-DC) or anti-His mAb (control DC) followed by co-culture with CD40L Tf. After 3 d, DC were harvested from and lysed, and cellular protein was subjected to western blotting for the indicated proteins. Data are representative of 3 experiments. (B) moDC were treated with HIV-1(+) serum (RNA copy number>400,000/ml) with or without pre-treatment by anti-DC-SIGN mAbs, as described in “materials and methods”, prior to coculture with CD40L transfectants as described in panel A, and cellular protein was extracted and subjected to western blotting for the indicated proteins. Data are representative of 3 experiments. (C) Human ASK1 (Ichijo H et al, Science 1997;275:90–94) was generated in pcDNA3 vector, as described (Won M et al, Cell Death and Differentiation 2010;17:1830–1841), and transiently transfected into HEK293 cells by lipofecatmine PLUS, according to manufacturer's instructions. After ≈36 hours, cell were lysed and subjected to Western blot assay with rabbit polyclonal anti-ASK1 Ab (Phospho-ASK1 (Thr845) antibody, #3765, Cell Signaling, USA). Results confirmed ASK1 expression with a molecular weight ≈160 kDa, which served as a positive control for p-ASK1 expression in Fig. 7A. Data are representative of 3 independent experiments. (TIF) [file ppat.1003100.s012.tif]
